# Fluorescence Sensing of Anions Like Adenosine 5ʹ‐Triphosphate With pH‐Gated Porous Dye‐Loaded Cerasomes

**DOI:** 10.1002/smll.75507

**Published:** 2026-08-31

**Authors:** Romane Vinot, Estela Climent, Maja Friedrich, Jérémy Bell, Knut Rurack

**Affiliations:** ^1^ Chemical and Optical Sensing Division Bundesanstalt für Materialforschung und ‐prüfung (BAM) Berlin Germany

**Keywords:** ATP, cerasome, fluorescence, gated sensor, pH

## Abstract

The development of gated systems capable of controlled opening of pores on demand represents an attractive way to control chemical functions in many fields of application. Here, we investigate the loading of various reporter dyes into non‐porous and porous cerasomes, a hybrid nanoscopic biomimetic material, and how pH‐dependent supramolecular adenosine 5′‐triphosphate (ATP) binding at the polyaminic gatekeepers anchored at the cerasomes’ pore outlets is influencing pH gating. Such organic‐inorganic nanovesicles are composed of a siliceous crosslinked lipid bilayer enclosing a liquid‐filled void. Preparation of porous cerasomes was achieved by inserting poloxamer block‐co‐polymers into the membrane during synthesis. To our knowledge, this is the first example of porous cerasomes and their use in gating applications. We successfully encapsulated or loaded reporter molecules into these objects, with the loading preference depending on their charge, hydrophobicity and chemical structure. The addition of molecular gates, responding to pH and/or anions such as ATP, allowed the pores’ controlled opening, thereby modulating proton influx, which can be read out via fluorescence response. Considering the corresponding pH sensitivity, dye loaded cerasomes are promising reagents for detecting ATP in relevant concentrations (0.02 to 1000 µm) with distinctly faster fluorescence responses as compared to standard gated mesoporous materials.

## Graphical abstract

The development of gated systems enabling controlled pore opening offers new opportunities for sensing applications. This study explores dye loading in porous and non‐porous cerasomes and pH‐dependent ATP‐triggered gating via polyamine anchors. Porous cerasomes were synthesized using block‐co‐polymers, enabling controlled proton influx and fluorescence‐based ATP detection with high sensitivity and rapid response.

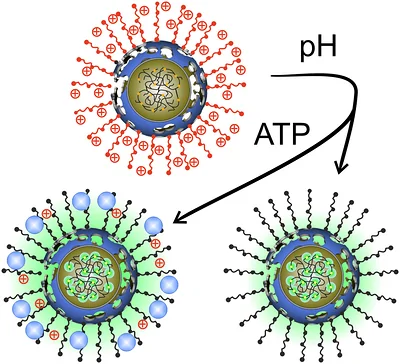

## 1 Introduction

For many years, hybrid materials have been a focus of research because they allow organic and inorganic chemical properties to be combined, often in ways that result in new features, making them attractive for applications beyond those of the individual materials [1]. Among the numerous materials and synthesis routes, hybrids prepared using silica as the base material via a sol–gel process are perhaps the most widely established, as this approach is compatible with a variety of other inorganic as well as organic materials and synthesis conditions, and offers access to a wide range of hybrid materials based on a wealth of commercially available silane precursors under standard laboratory conditions [2]. However, to prepare dedicated hybrid sol–gel materials that go beyond solid particles or functionalized surfaces, molecular self‐assembly steps are crucial. Indeed, the properties of nanohybrids depend on their nanoscopic structure, especially the structure at the interface between the organic and inorganic components. Nevertheless, the structure of this interfacial region is complex to control, and in most cases the final material is largely or entirely composed of the inorganic part only, while the organic part is used as a (sacrificial) template or functional coating. To prepare materials with a truly hybrid skeleton, we became interested in materials composed of amphiphilic molecules with a covalent bond between an organic and an inorganic moiety. One such class of hybrid objects we found particularly intriguing are cerasomes (Figure 1) [3, 4, 5, 6]. These hybrid vesicles are prepared through a combination of a sol–gel procedure and the self‐assembly of lipidic organotrialkoxysilanes to form bilayer‐type vesicles expressing siliceous surfaces. Compared with conventional all‐organic liposomes, the inorganic siloxane framework provides them with remarkable stability, particularly against surfactants [6], and allows subsequent surface tailoring with silane derivatives. Moreover, liposomes tend to aggregate and fuse into larger vesicles [7], which presents a genuine problem for medical applications like drug delivery [8] or in vivo sensing. This vesicle fusion cannot happen with cerasomes due to the rigid surface formed by silica crosslinking. Therefore, cerasomes combine all the qualities of liposomes like excellent biocompatibility and, presumably, good biodegradability [9], enabling an easy elimination from the body after use, with the advantage of better stability and the possibility for versatile surface tailoring. In the context of our research on the gated release of small molecules [10, 11, 12], we became interested in porous cerasomes for sensing applications and developed a flexible nanoscopic molecular hybrid system with polyanion‐modulated gating functions that can be controlled by both pH and the presence of anions such as 5′‐adenosine triphosphate (ATP). The detection of physiologically relevant anions such as the phosphates of nucleosides is of eminent importance in biochemical analysis and diagnostics [13, 14]. Among the nucleotides, ATP has been the primary object for the development of water‐based selective probes [15, 16]. Aside from providing energy to all living organisms on Earth, ATP is engaged in a variety of biological processes, including DNA replication and serves as a cofactor in several enzymatic processes as well as hormonal and neurological functions [17]. Interestingly, ATP can be used as a marker for the presence of microbial loads in various environments like water, surfaces, or on hands [18, 19]. The ability to detect ATP traces with new sensory assays based on a signal amplification mechanism would therefore be of great interest to detect microbial loads or contamination sensitively and quickly at a point‐of‐suspect, e.g., in hospital or home hygiene. While there are several reliable techniques for measuring ATP levels, like HPLC and bioluminescence, their complexity or high limits of detection limit their applicability [20, 21, 22, 23, 24]. Gated indicator systems, known for their high sensitivity [25] based on sub‐micron‐sized cerasome hybrids could thus fill a capability gap where the above methods are not applicable or not sensitive enough. Compared to gated materials based on mesoporous silica nanoparticles of MCM‐41 or SBA‐15 type, these new objects would also have better biocompatibility and would suffer less from sedimentation.

**FIGURE 1:**
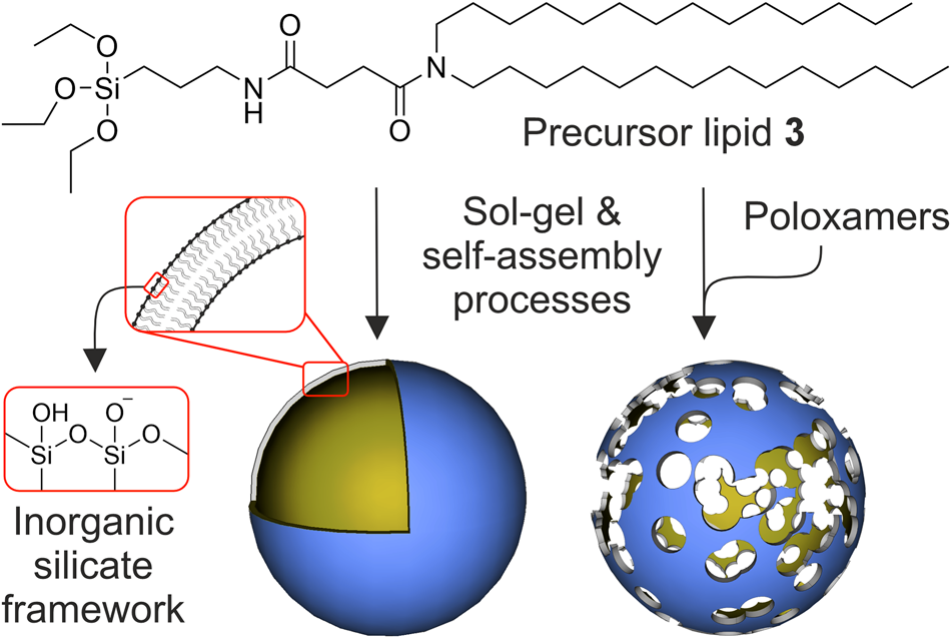
Formation of conventional non‐porous cerasomes and porous cerasomes with siliceous surface layers from a specific precursor lipid **3**; crosslinked silane head groups form the inner‐ and outermost layers.

## 2 Results and Discussion

### 2.1 Synthesis of the Cerasome Precursor

The cerasome precursor was prepared following literature procedures, employing a modified protocol reported by Kikuchi et al. [3, 4, 5, 6, 26, 27]. Since none of the cerasome‐forming lipids was commercially available, the more straightforward route for the preparation of *N*‐[*N*‐(3‐triethoxysilyl)propylsuccinamoyl]dihexadecylamine **3** was followed (Figure S1). First, *N*, *N*‐dihexadecylamine **1** was obtained by alkylation of hexadecylamine with hexadecyl bromide in the presence of potassium carbonate. In the second step, *N*, *N*‐dihexadecylsuccinamic acid **2** was obtained by coupling of **1** with succinic anhydride. Lipid precursor **3** was obtained after activation of the carboxylic acid moiety with dicyclohexylcarbodiimide and *N*‐hydroxysuccinimide through addition of 3‐aminopropyltriethoxysilane (APTES), affording the final product with a good yield of 77%.

### 2.2 Preparation and Characterisation of Non‐Porous Cerasomes

Non‐porous cerasomes were prepared by self‐assembly and crosslinking of the lipid **3** (Figure S2). For this purpose, various mixing and sonication times were tested for reproducible preparation of the particulate objects. It was observed that the mixing time has a negligible influence on the preparation of cerasomes. However, the solution temperature during the sonication step was found to be a key parameter. Whereas at 25°C, no cerasomes were obtained, at 70°C the solution began to evaporate, and the micelles were destroyed. It was found that 55°C was the best sonication temperature, this temperature presumably initializing the phase transition from micelles to cerasomes through initial silanol head group crosslinking. Additionally, power and time of sonication were also found to be important parameters. When the sonication time was too long, the pre‐cerasomes (micelles without fully crosslinked silanol layers) were destroyed, but when it was too short, the cerasome formation did not take place. Therefore, time and power of sonication are closely related; if one is increased, the other one must be decreased. After the final step of sonication for 5 min with 75 W acoustic power at 55°C, the obtained solution was stored in a refrigerator for some days to allow the silanol network to fully crosslink. To speed up the reaction, the crosslinking step can also be done at room temperature. The cerasome formation kinetics were qualitatively checked with a flow cytometer (Figure S3). Although the cerasomes themselves are too small (diameter determined to 200–300 nm by dynamic light scattering (DLS) and scanning electron microscope (SEM), see Figure 2 and Figure S4) to be detected with conventional cytometers, their so‐called swarm detection in a dot plot of side scatter (SSC) vs. forward scatter (FSC) allows to trace their formation rate in a facile manner (Figure S3). Zeta potential measurements were also invoked for cerasome characterization, revealing that, when the preparation is carried out at pH 3, the zeta potential of the cerasomes is moderately positive (between +15 and +25 mV), largely avoiding aggregation or fusing during the preparation process when a fully covering silica surface layer is not yet present (small peak between 1–10 µm in Figure S4). Also, the subsequent basification to stop the reaction does not influence the morphological properties of the objects formed, i.e., fusion and aggregation is found to be minimal while the hydrodynamic diameter has been determined to 343 ± 26 nm by DLS, with a polydispersity index PDI = 0.34 (Figure S4). After full cross‐linking of the cerasomes’ inside and outside silica surface, zeta potential measurements were also performed at various pH (Figure S5). At pH 4, the zeta potential became negative (−25 mV), already lower than the zeta potential of non‐crosslinked lipid precursor micelles (−5.5 mV). When the sample is basified to pH >7, the value of the zeta potential is progressively decreased to −45 mV which makes it possible to distinguish between micelles (−10 mV at pH 11.5) and cerasomes (−45 mV at pH 11.5), proving siloxane network condensation. Size exclusion chromatography (SEC) was performed to remove micelles and precursor lipids that did not react. After the purification, the PDI of the suspended cerasomes decreased to 0.20 (254 ± 19 nm) compared to 0.34 (343 ± 26 nm) before SEC, which agrees well with previous reports [28] and indicates that the size selection through SEC is applicable for objects such as cerasomes (Figure S4). After clean‐up, the cerasomes were stored in a refrigerator for up to 4 years without any sign of degradation of the vesicles. Purified cerasomes were also characterized by Scanning Electron Microscopy (SEM) (Figure 2) and, like reported before, it was observed that the cerasomes have a spherical structure with an approximate diameter of 148 ± 14 nm and are hollow.

**FIGURE 2:**
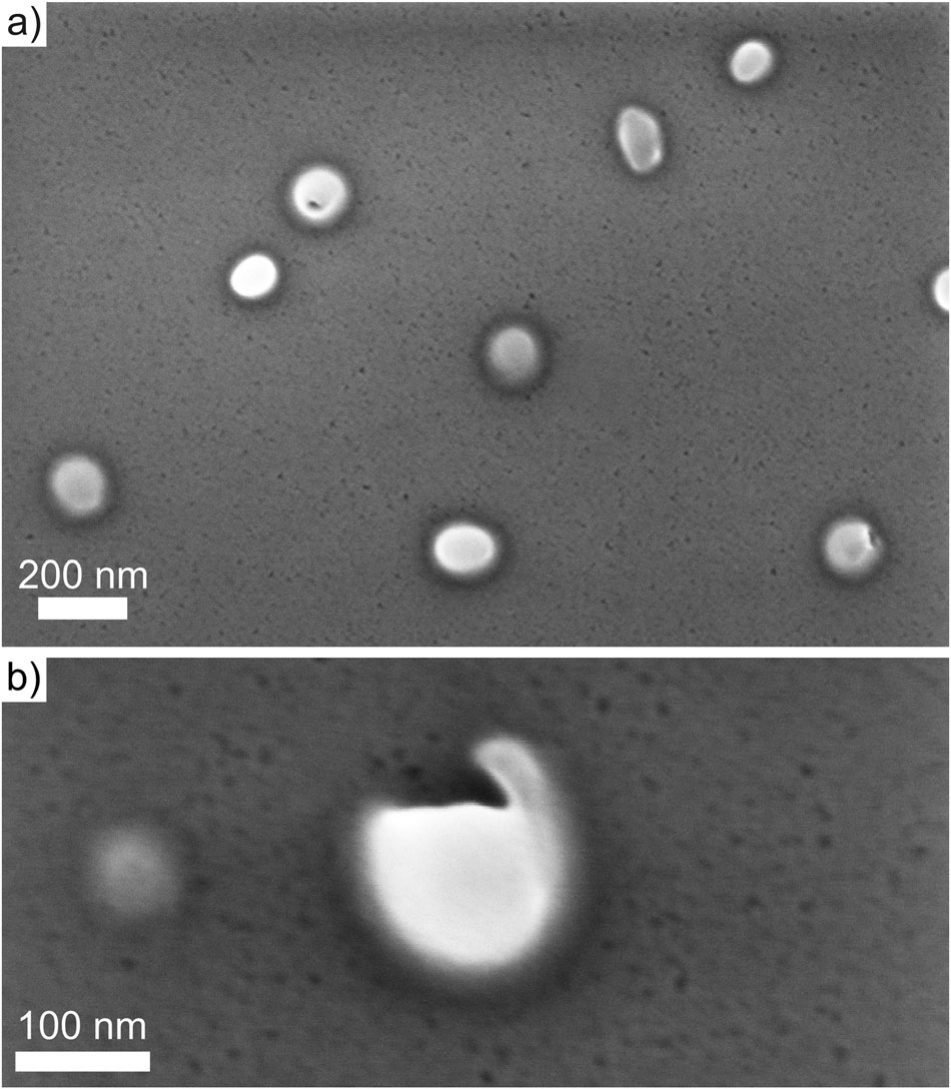
(a) and (b) SEM images of non‐porous cerasomes.

### 2.3 Dye Encapsulation in Non‐Porous Cerasomes

Since the encapsulation of small organic molecules or organic macromolecules into cerasomes is of great interest for delivery and sensing, several dyes were selected to simulate neutral, positively, or negatively charged organic compounds as well as small and large (macro)molecules as cargo. The dyes were: rhodamine 6G chloride (**R6G**), rhodamine B chloride (**RhB**), sulforhodamine B sodium salt (**SRB**), safranin O chloride (**SfrO**), tris(bipyridine)ruthenium(II) dichloride (**RBP**), nile blue chloride (**NB**), 6‐carboxyfluorescein (**6‐FAM**) and two FITC‐labelled dextran polymers with MW ≈ 4000 g mol^−1^ (**FITC4000**) and MW ≈ 70000 g mol^−1^ (**FITC70000**). The last two labelled polymers were chosen to be able to monitor membrane permeation or the opened/closed state of the pores of the vesicles. Such macromolecule‐bound dyes are weakly fluorescent at high concentration in a confined space due to the inner filter effect, dye‐dye interactions (e.g., homo‐energy transfer) or reabsorption [29], but show a pronounced fluorescence enhancement when released from vesicles into a solution, accompanied by dilution, resulting in the unfolding of the packed branched polymer and the singulation of the dyes. Besides the massive release of cargo from gated indicator release systems [30], such inherent amplification phenomena present another favourable feature for vesicle‐ or cerasome‐based sensing schemes to reach low detection limits and provide high signal‐to‐noise ratios. The dyes were added at concentrations of 4.0 g L^−1^ (except for **FITC70000**: 40 g L^−1^) to solutions of the lipid **3** at pH 3 before the formation of non‐porous cerasomes, to study cerasome formation and encapsulation capabilities simultaneously. The addition of 5% v/v of ethanol into the aqueous mixture (W5E) was also tested to assess the encapsulation of organic material that would require an organic co‐solvent for sufficient solubility [31, 32]. After cerasome formation and crosslinking, followed by SEC to remove any free dye, the encapsulation efficiency was assessed via the dyes’ absorption spectra accompanied by fluorescence and flow cytometry analysis (Figure 3a–c, Figures S6–S9, Table S1). Assuming complete removal of excess dye and negligible passive leaching, the absorption spectra of undiluted or diluted solutions of cerasomes were used to quantify the overall dye concentration in the respective cerasome suspensions via the molar absorption coefficients of the dyes, yielding the encapsulation efficiency (*EE*_abs_) of the cerasomes for the different dyes. In addition, the emission spectra of these solutions, and especially the shift of the emission maximum as a function of the concentration of the dyes confined in a cerasome due to reabsorption, provide further valuable information of the dye's environment and/or concentration inside the cerasomes. Regarding particle dimensions, the average diameter of the loaded objects remained approximately the same as that of the plain cerasomes filled only with solvent, i.e., 269 ± 89 nm.

**FIGURE 3:**
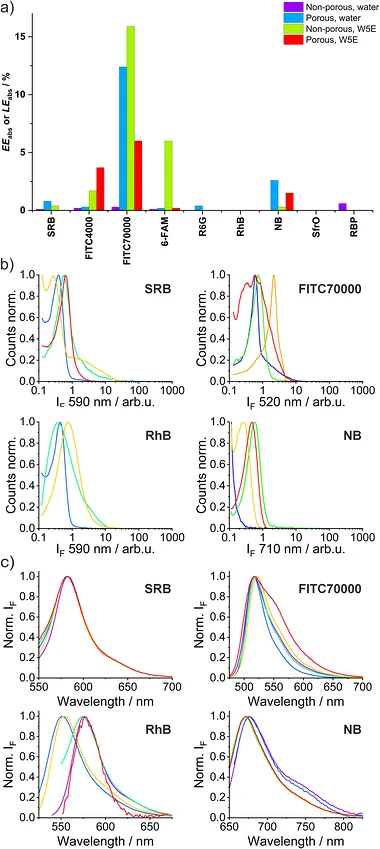
(a) Approximation of the encapsulation efficiencies *EE*_abs_ into non‐porous cerasomes and loading efficiencies *LE*_abs_ into porous cerasomes of the dyes as derived from spectroscopic measurements; (b) Flow cytometry emission histograms and (c) normalized emission spectra from the dyes in solution and the obtained cerasomes (purple lines: dyes in solution; blue lines: non‐porous cerasomes formed and loaded in pure water; yellow lines: porous cerasomes loaded in pure water, green lines: non‐porous cerasomes formed and loaded in W5E; red lines: porous cerasomes loaded in W5E).

Table S1 reveals that the encapsulation of prototropic neutral/anionic dyes (**FITC4000**, **FITC70000**, **6‐FAM**) in non‐porous cerasomes in W5E represents the most efficient loading scenario on average for all the dyes and approaches. At the same time, encapsulation of non‐porous cerasomes in neat water was the least efficient, with only **FITC70000** being loaded with an encapsulation efficiency (*EE*_abs_) of 0.3%; note that the porous cerasomes will be discussed in detail in the next section below. In this scenario, encapsulation was negligible for **6‐FAM**, **FITC4000** and **SRB** with *EE*_abs_ <0.2%. In W5E, encapsulation for **SRB** was somewhat improved, but significantly higher only for the three fluorescein derivatives with up to 6.0% for **6‐FAM** and 15.9% for **FITC70000**. The encapsulation of permanently cationic dyes in non‐porous cerasomes was generally low, with ≤0.1% for **R6G** and **RhB** in both media, for **NB** in water and **RBP** in W5E. While the latter two showed slightly higher loadings in the other medium, i.e., 0.6% for **RBP** in water and 0.3% for **NB** in W5E, essentially no loading could be confirmed for **SfrO** in either medium.

The absorption and emission spectra of the dyes in the various scenarios shown in Figure 3c, Figures S6–S8 and Table S1 support these results. For example, the fluorescence maxima of the anionic and most cationic dyes differed by ≤ ±3 nm when encapsulated in non‐porous (and porous) cerasomes in water and in W5E. For **SRB**, the virtually unchanged absorption and emission spectra suggest that the small amounts encapsulated in non‐porous cerasomes in W5E are mainly isolated dye molecules that do not aggregate or interact significantly with the hydrophilic or hydrophobic regions of the crosslinked objects. **RBP**, which is incorporated in smaller amounts into non‐porous cerasomes in water, exhibits similar behavior; in W5E, there is a slight narrowing of the Stokes shift, which could indicate an attraction to the non‐polar region of the cerasomes or an interaction with the anionic silanolate groups of the silica network of the cerasomes rather than with Cl^−^ counterions. **NB** and **R6G** show virtually unchanged emission spectra. For **NB**, the shift in the emission maximum also correlates with the increase in the *EE*_abs_ rate (Figure S8). The rich band structure and shape of the bands observed for **6‐FAM** in absorption suggest that prototropic equilibria are still active, which is also reflected in the emission spectra [33]. For **FITC4000** and especially **FITC70000**, these features are also visible to some extent.

The only dye that shows consistently different behavior when encapsulated in non‐porous cerasomes in water is **RhB** (Figure S10a,b). Before discussing the spectroscopic features observed in the experiments it is helpful to recall the photophysics of **RhB**. **RhB** can exist in three different forms, the spiro‐type lactone (**RhB_L_**), the zwitterionic **RhB** base (**RhB_B_**) and the intrinsically cationic **RhB** acid (**RhB_A_**), see Figure S11. For the neat dye, depending on pH, either **RhB_B_** or **RhB_A_** exist in polar protic solvents (e.g., alcohols) and water. These species show the typical polymethinic absorption and emission bands with maxima at 542 and 565 nm (**RhB_B_**) as well as 553 and 576 nm (**RhB_A_**) [34, 35]. In aprotic solvents, if the lactone form is used, **RhB_L_** dominates, leading to significantly blue‐shifted, less intense absorption bands with maxima at 310–315 nm for solvents from cyclohexane to acetonitrile whereas the emission is strongly solvent dependent in both intensity and maxima, ranging from 400 nm in cyclohexane (weak) to 524 nm in MeCN (moderate) [36]. If a **RhB** salt is used, either **RhB_A_** or **RhB_B_**, the typical polymethinic characteristics are observed with absorption at ca. 555 nm and emission at 583 and 580 nm in nitriles or chloroform [37, 38]. Figures S6 and S7 reveal the following characteristics: encapsulation of **RhB** into non‐porous cerasomes in water leads to absorption and emission maxima at 523 and 551 nm while for water‐ethanol mixtures, these bands are found at 557 and 575 nm. Indeed, as explained above, due to possible exclusive loading of this dye in its **RhB_B_** form into the hydrophobic lipid interlayer (Figure S10c), emission quenching and a wavelength shift could occur: cerasomes loaded with **RhB_B_** exhibited green emission (552 nm), while free **RhB_A_**—or **RhB_B_**, depending on the pH of the aqueous solution—showed orange emission (576 nm). Because this shift could not be caused by the conversion of the dye into the non‐polar spiro‐lactone form, as the latter is only UV‐absorbing and non‐emissive, we recorded the spectroscopic properties of **RhB** in various solvents and correlated them with empirical solvent polarity scales, to assess whether these unique features can be tentatively ascribed to a possible confinement of **RhB** in its xanthene form in the hydrophobic interlayer (Figure S10d). Indeed, when the emission maximum of **RhB** in various solvents is plotted against the empirical solvent dipolarity and polarizability scale *SPP*_N_ [39], a reasonable correlation is obtained, which allows to deduce *SPP*_N_ values of 0.562 and 0.595 for **RhB** in both types of cerasomes (see below for details on porous cerasomes). Such values are characteristic for hydrophobic environments constituting of linear (*SPP*_N_ = 0.562 for *n*‐decane) [39] or cyclic alkanes (*SPP*_N_ = 0.590 for cyclooctane, 0.601 for *cis*‐decaline) [39] and might even hint at the order of the long tails in the cerasomes’ interlayer. Why the xanthene form is preferred to the spiro‐lactone form in such a scenario is not entirely clear but might be connected to the spiro form being in a rigid orthogonal arrangement and/or that the net positively charged xanthene moiety of **RhB** might be aligned toward the net negative (inner and outer) silica rims of the cerasomes. This phenomenon was not observed in the presence of ethanol, which is consistent with the lower loading efficiency. Furthermore, if **RhB** is only encapsulated in the hydrophobic region, the overall loading efficiency is very low as is evident from Table S1. Fluorescence anisotropy measurements did not reveal a significant increase in anisotropy compared with **RhB** in aqueous solution (Figure S10e). This spectroscopic feature appeared advantageous for fast cerasomes cleanup: The ELISA plate containing the collected fractions after chromatography could be illuminated under a UV‐lamp (*λ*_exc_ = 365 nm) to visibly determine the wells containing loaded cerasomes.

Following the spectroscopic studies, we investigated the loaded cerasomes by flow cytometry to assess whether single or multiple populations are present. As we have discussed above, the cerasomes themselves are too small to be visible as single objects with our cytometers, but for the dilute solutions that exist after loading, crosslinking and washing, the histograms that are obtained in the respective fluorescence channels allow to draw conclusions about the dispersion state of the objects. The bottom row in Figure S9 shows that the comet tail type behavior is not found after the loading and cleanup procedure, entailing a dilution of the cerasome concentration and retrospectively supporting the above explanation of multi‐object events leading to the shapes of the dot plots in Figure S3 and not cerasome aggregation. Furthermore, the two dot plots in Figure S9 exemplarily show that the radial distribution of nanoparticles contained in Milli‐Q water (middle panel) is expanded on both axes, but somewhat more on the SSC axis for the cerasome‐containing solution (right panel), indicating the presence of small, more strongly scattering objects. This is very similar to the changes in Figure S3 as cerasome formation progresses and is thus also attributed to the presence of cerasomes. Taking into account that the dye solutions themselves only show a negligible fluorescence, as is exemplarily shown in Figure S9, bottom row, left panel, the shape of the histograms recorded for the loaded cerasomes can provide an indication about the monodispersity and the number of populations formed.

Figure S9 reveals that when non‐porous cerasomes are loaded and crosslinked in water, except for **FITC70000**, only rather narrow monomodal populations are found, indicative of the formation of well‐defined objects. For **FITC70000**, a certain tailing toward higher fluorescence signals is observed, which can possibly result from different degrees of intramolecular quenching due to conformational changes in the dye‐labelled dextran. This is also supported by a comparison of the behavior of **6‐FAM**, **FITC4000** and **FITC70000** in non‐porous cerasomes in the two media used, water and W5E: whereas the blue and the yellow curve are largely overlapping for **6‐FAM** in Figure S9, the fluorescence is generally higher in the presence of the small amount of organic solvent for **FITC4000** and **FITC70000** in W5E (yellow vs. blue curve, Figure S9). For the anionic (**SRB**) as well as the cationic rhodamines (**R6G**, **RhB**), single rather narrow populations are found for non‐porous cerasomes prepared in water, yet much broader populations when prepared in W5E. This behavior is tentatively ascribed to specific solvation effects and the possibility of the dye molecules to interact with the various hydrophilic and hydrophobic parts of the cerasomes. For **NB** and **SfrO**, the fluorescence histograms correlate well with the loading efficiencies reported in Table S1, and for **RBP**, the differences are difficult to interpret in view of the other data. For the FITC dextran dyes, homogeneous encapsulation could be achieved as revealed by flow cytometry with the expected quenching of the emission for **FITC4000** and **FITC70000** [40, 41, 42].

To resume, **FITC4000** and **FITC70000** showed efficient loading into non‐porous cerasomes, especially in the presence of low amounts of ethanol (W5E), with **FITC70000** reaching >60% encapsulation. In contrast, small cationic dyes, such as **SRB**, **R6G, RhB, SfrO, RBP** displayed very low loading efficiencies with minimal spectral changes, reflecting weak or even repulsive interactions with the cerasome matrix. Only **NB** could be loaded at almost 10% into porous cerasomes. Interestingly, **RhB** exhibited specific distinct spectral shifts when loaded, producing blue‐shifted emission, useful for the identification and separation of loaded cerasomes.

### 2.4 Preparation and Characterisation of Porous Cerasomes

One of the potential applications of cerasomes would be as nano carriers for drug delivery, diagnostics or theranostics, for instance in combination with a (bio)chemically triggered gating function. For such a purpose, it is necessary to efficiently encapsulate small organic molecules inside the cerasomes but also to quantitatively release them once the gates are opened. Triggered drug release from cerasomes has so far only been realized with physical stimuli such as UV irradiation [43], temperature changes [44], or redox potential changes [45]. Based on our experience in chemically targeted release of indicators from gated mesoporous hybrid silica materials [10, 11, 12], for which the silica scaffold is first prepared, then the cargo loaded and finally the gating (bio)chemistry applied, we also opted for the formation of porous cerasomes and their subsequent modification by loading and gating here, to allow controlled released upon external stimuli without destruction of the crosslinked vesicles [46]. Interestingly, it was previously reported by Johnsson et al. [47], that low molecular weight poloxamer macromolecules could induce defects and leakage from liposome membranes. Thus, we decided to introduce pores into the cerasome membrane by adapting the poloxamer approach while still allowing for the crosslinking of the membrane, screening for an optimized mixture of alkoxysilanes and poloxamers. The idea was to create sacrificial rods through poloxamer insertion into the membrane, which can then be removed through mild chemical treatment.

Cerasomes were thus synthesized in the presence of different types of poloxamers, i.e., with Pluronic P‐123 (MW ≈ 5800 g mol^−1^), Pluronic F‐127 (MW ≈ 12600 g mol^−1^) and Pluronic F‐68 (MW ≈ 8350 g mol^−1^) (Figure 4a), employing either 5 or 50% wt of poloxamer and the precursor lipid **3** in water at pH 3. First, it was observed that the formation of the siloxane network took on average longer in the presence of the poloxamer (approx. 2 weeks). The poloxamers hinder silane condensation to some degree due to their insertion into the vesicle's lipid bilayer membrane. As this procedure is rather impractical, we used another approach and added auxiliary agents to the poloxamer/precursor **3** mixture, i.e., iron oxide nanoparticles (IONPs) and rhodamine 6G (**R6G**) [48, 49], finally reducing cerasome formation time to 4 d. Utilizing this strategy, the faster kinetics of membrane formation in the initial stage (before crosslinking) is attributed to the increased fluidity of the vesicles’ membranes in the presence of IONPs as described for liposomes [48, 49]. Similarly, **R6G** is assumed to further stabilise the freshly formed vesicles [50].

**FIGURE 4:**
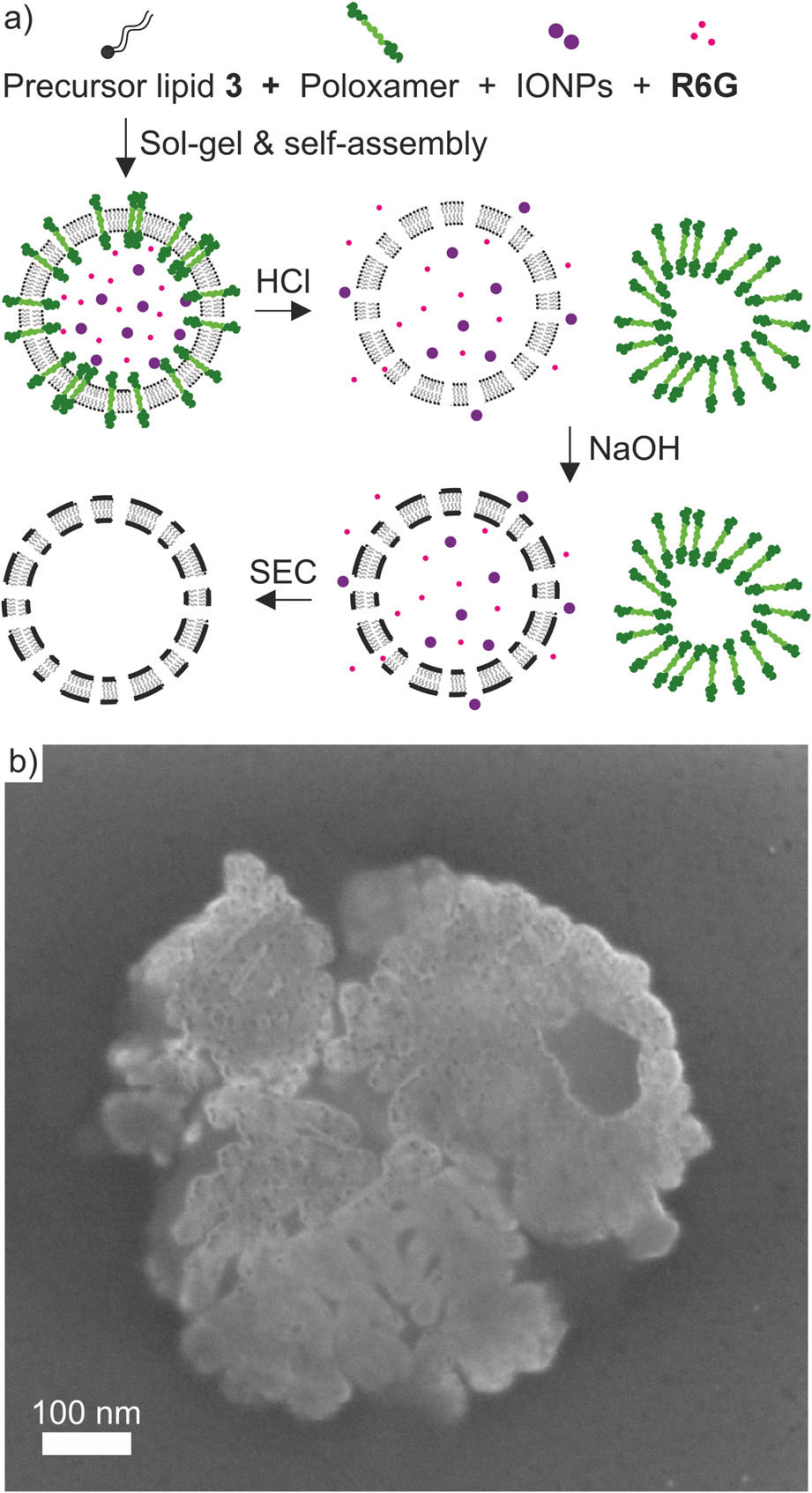
(a) Preparation of porous cerasomes from precursor lipid **3**, poloxamer, IONPs and **R6G** by self‐assembly, cleaning by acidic treatment, basification with full crosslinking of the silanol heads, and size exclusion chromatography (SEC); (b) SEM image of a porous cerasome after clean‐up. This picture of a slightly oversized cerasome was selected for clarity and pores viewing, attempts of imaging of smaller sized porous cerasomes induced their destruction due to intense irradiation power coupled to the vacuum conditions (see Figure S14).

As in the case of the non‐porous cerasomes, the solution was basified after the assembly and crosslinking phase. DLS analyses showed that mostly aggregates and micelles of poloxamer with an average diameter of 1632 ± 195 nm have been formed in the reaction solution. However, after treatment with HCl (1 m) in ethanol to dissolve the poloxamer and SEC to separate the cerasomes from residual poloxamer micelles and other molecules present in the solution, DLS analyses showed the presence of objects with a significantly reduced average diameter of 226 ± 20 nm (Figure S12), which was attributed to successful cerasome formation.

After purification, **R6G** (0.25 g L^−1^) was used as an indicator in combination with flow cytometry to evaluate the possibility of dye loading into the porous cerasomes through post‐encapsulation, thereby providing information on the presence of pores in the cerasome membrane and allowing for their tailoring through the iterative use of poloxamers of different type and concentration (Figure S13). Emission windows at 520 and 590 nm were selected in flow cytometry, corresponding to **R6G**’s emission spectrum. Various types of cerasomes were added to the dye solutions and mixed overnight by stirring, whereafter the mixtures were directly analysed to check for loading. As a control, the entire process was also carried out for dyes and non‐porous cerasomes. The histograms acquired for the latter gave low signals mainly due to scattering, being virtually identical to the neat dye in solution. When the cerasomes were prepared using poloxamers without additives, weak fluorescence signals were also measured, suggesting that no **R6G** could be loaded into the cerasomes and that, accordingly, no pores had formed in the cerasome membrane. However, when a low molecular weight poloxamer (Pluronic P‐123) was used for fabrication at 50% wt in combination with IONPs and **R6G**, more objects with a higher fluorescence in both 520‐ and 590‐nm channels were counted, indicating the successful loading of **R6G** into the cerasome and, consequently, the presence of pores.

SEM image analysis of the porous cerasomes showed objects with an almost spherical shape with an average diameter of 302 ± 60 nm, random pores from 10 to 100 nm and a hollow structure (Figure 4b, Figure S14a,b). Moreover, irregularly crosslinked structures of the silanol network were also observed. Due to their greater fragility, most objects exploded in the vacuum, revealing a snowflake‐like crosslinking as it was presumably induced by the presence of the poloxamer. Interestingly, some residual cerasomes of a rather large size (up to 10 µm) could also be observed (Figure S14c,d). On the surface of these objects, the crosslinking pattern can be clearly seen with a mixture of propagating and branched features, hindered by the presence of poloxamer, revealing defects (pores) in the final membrane.

By obtaining pores, we were subsequently able to demonstrate a proof‐of‐concept for the use of porous cerasomes as gated particles for applications in controlled delivery and sensing. For this purpose, we decided to attach the polyamine *N’*‐(3‐trimethoxysilyl) propyl diethylenetriamine (**N3**) on the surface of the porous cerasomes to obtain a model of a polyanion‐modulated pH‐ and anion‐sensitive molecular gate, analogous to systems described in the literature for mesoporous MCM‐41‐type silica materials (Figure 5) [51]. In order to use porous cerasomes as drug‐ or probe‐delivering particles, selective pore opening and closing of the pores must be achieved. After the silane **N3** was condensed onto the surface of the porous cerasomes, an acidic medium (pH 3) caused pore closure through a mixture of electrostatic repulsion of the positively charged ammonium chains and electrostatic attraction of these chains by counterions. Later, a basic medium (> pH 7) would cause the pores to open, as the polyamine gating molecules would adopt a linear arrangement in their neutral state.

**FIGURE 5:**
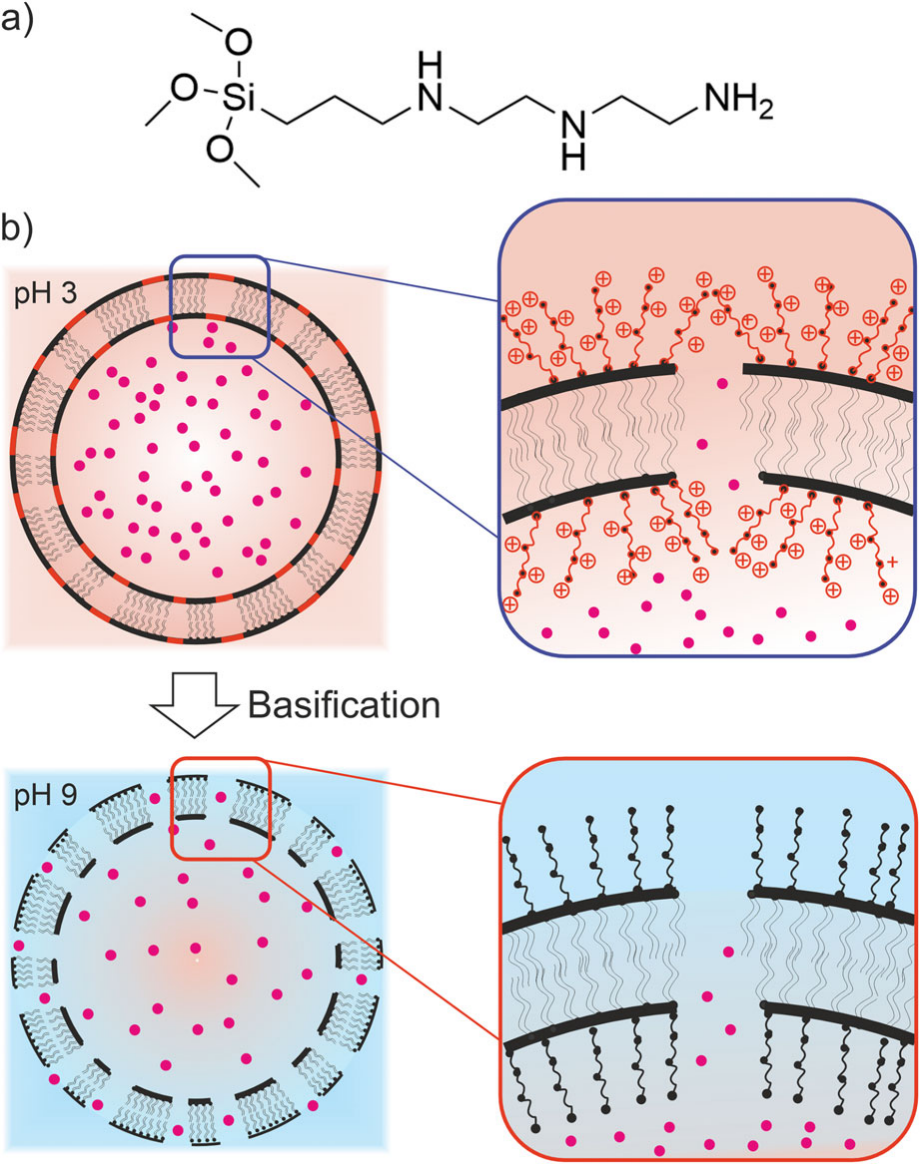
(a) Structure of *N’*‐(3‐trimethoxysilyl) propyl diethylenetriamine (**N3**); (b) Scheme of the polyanion modulated pH‐dependent gating mechanism on the surface of dye‐loaded porous cerasomes with rapid gate opening and pH equilibrium within the cerasome void and slower release of the loaded cargo; colour code: orange = lower pH, blue = higher pH.

Various condensation reaction times were tested to graft **N3** groups to the siliceous surface of the porous cerasomes, namely 12, 3 and 1 h, the cerasomes being labelled **C‐12 h**, **C‐3 h** and **C‐1 h**, respectively. After completion of the reaction with **N3** the solution was acidified to protonate its primary and secondary amino groups, stopping the condensation reaction, and closing the cerasomes’ pores. SEC was then performed to remove unreacted **N3** and free dye in solution (Figure S15a). For optimisation of the protocol, **RhB** was loaded into the cerasomes before addition of the **N3** gates. Afterward, the pores were closed by acidification. Indeed, like for the non‐porous cerasomes above, **RhB** shows the same consistently different behavior when loaded into porous cerasomes in water, exemplified by the shapes of the spectra in Figures S6 and S7. **RhB** is presumably encapsulated in monomeric form within different compartments, as revealed by the aforementioned strong blue shift in absorption (530 nm) and emission (553 nm) observed when the dye is loaded into porous cerasomes in pure water rather than in W5E. As explained above, such a shift is most likely connected to **RhB** residing in its xanthene form in the hydrophobic lipid interlayer (Figure S10), as this phenomenon was not observed in the presence of ethanol. For **C‐1 h** cerasomes, it was observed that no dyes were encapsulated, since only the fluorescence of the free dye was observed under UV irradiation. Therefore, only **C‐3 h** and **C‐12 h** were studied in the next step. The collected gated and loaded cerasomes were submitted to basification to induce gate opening. For **C‐12 h**, no changes were observed, indicating a complete and permanent closure of the pores, which is likely due to a multilayer crosslinking of **N3**. However, **C‐3 h** showed variations in its fluorescence spectra when changing the pH of the medium. The pore opening induced rapid quenching of the dye emission (Figure 6). This indicates that the pH change is too fast for **RhB_A_** exiting the bilayer into the cerasome void or the external medium, so that it is presumably converted into **RhB_L_** (Figure S11) and remains locked in the cerasome membrane [52]. The control experiment with non‐porous cerasomes loaded with **RhB** and placed into a basic environment also showed no significant response.

**FIGURE 6:**
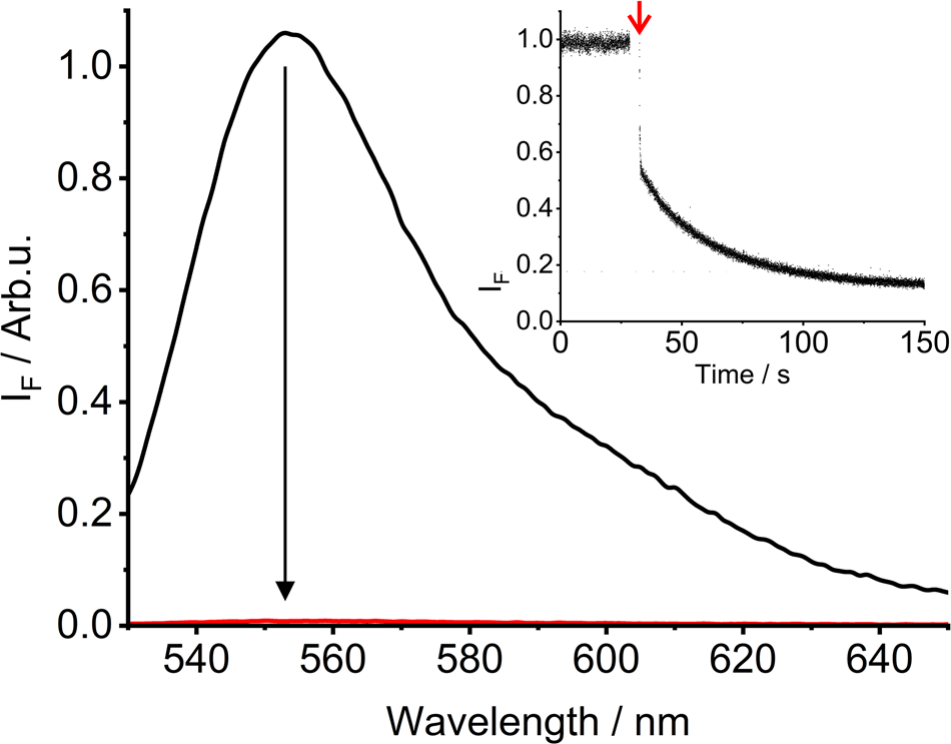
Emission spectra of a solution containing **C‐3 h** (**RhB**‐loaded porous cerasomes with polyanion‐modulated pH gates) before basification (black line) and upon basification of the medium (red line); Inset: Emission of the solution at 552 nm vs. time; red arrow denotes NaOH addition (*l*_exc_ = 520 nm).

Subsequent investigation concerned the loading of various dyes in the porous cerasomes. As described for **RhB**, those dyes were added with concentrations of 4.0 g L^−1^ to a suspension of porous cerasomes in Milli‐Q water or W5E and mixed for 24 h to ensure complete diffusion inside the cerasome cavity. Subsequently, the **N3** gate was grafted on the surface of the particles by a condensation reaction for 3 h. After loading and gate closing (pH ≤ 3), size exclusion chromatography using MilliQ at pH 3 as eluant was used to remove free dyes and excess of **N3**. To ensure that the pores remain closed, the cerasomes were kept in suspension at pH 3 at 4°C. Under such conditions, all prepared batches showed remarkable stability over time (> 1 y). The loading efficiency was controlled via the dyes’ absorption bands and verified by emission signals recorded with a fluorometer and flow cytometry (Figure 3a–c) (Figures S6–S9, Table S1).

With respect to the loading characteristics of all the dyes used in this work, the average loading was slightly higher for porous cerasomes in water than in W5E, with the general differences between anionic and cationic dyes remaining unchanged to those observed for the non‐porous cerasomes above (Table S1). **RBP** could not be loaded into porous cerasomes, **SfrO** only in small quantities in W5E, **RhB** only in small quantities in water and **R6G** at approx. 0.4% in water. Only **NB** was encapsulated better in both media, i.e., at 1.5% in W5E and 2.6% in water. Moreover, the loading of anionic dyes was also inefficient for **6‐FAM** in both media (≤0.2%) and only somewhat increased for **SRB** in water (0.8%). High loadings were thus mainly observed for the dye‐labelled dextrans **FITC4000** and **FITC70000**, but only in the latter case in significant amounts in both media (12.5% and 6.0%); **FITC4000** was only incorporated to a significant extent in W5E (3.7%).

Like for the non‐porous cerasomes above, the absorption and emission spectra of the dyes in the various scenarios shown in Figure 3c, Figures S6–S8 and Table S1 support these results. As for the non‐porous cerasomes, the fluorescence maxima of the anionic and most cationic dyes differed by ≤ ±3 nm when encapsulated in porous cerasomes in water and in W5E. The virtually unchanged spectra in the case of **SRB** suggest that **SRB** is also encapsulated mainly as isolated molecules in porous cerasomes in water without pronounced interaction with the hydrophilic or hydrophobic regions of the cerasomes. For **SfrO**, the narrowed Stokes shift when encapsulated in porous cerasomes in W5E suggests a preferential interaction of the dye with the hydrophobic regions of the cerasomes. The pronounced formation of new absorption bands when encapsulated in porous cerasomes in W5E suggests dimerization or self‐assembly happen to some extent for **NB** and **R6G** under these loading conditions. Also, for the porous cerasomes, the rich band structure and shape of the bands observed for the fluorescein derivatives **6‐FAM**, **FITC4000** and **FITC70000** in absorption hint at the presence of a mixture of anionic and neutral species within the cerasomes. This corresponds well to the different forms of fluorescein with p*K*_a1_ = 2.1 and p*K*_a2_ = 4.3 in aqueous solution [33]. Since three net neutral forms of fluorescein are known, an uncharged UV‐absorbing lactone form, a yellow zwitterionic form and an also uncharged reddish *p*‐quinoid form, with the lactone and quinone being more lipophilic than the zwitterion, the present spectra indicate the presence of the *p*‐quinone [33, 53]. This suggests that there are less polar regions in the encapsulated **FITC70000** compared to the **FITC4000** that can easily accommodate the quinoid form. As would be expected, the fluorescence spectra are dominated by the moderate and high fluorescence of the small amounts of monoanion (and possibly also dianion) present in the solution, which have a *Φ*_f_ = 0.3 (monoanion) and 0.93 (dianion), compared to a *Φ*_f_ ∼0 for neutral fluorescein (Figure S18) [33].

In line with the previous section, the loaded porous cerasomes were subsequently analyzed with flow cytometry with regard to their population formation behavior (Figure S9). Also for this type of cerasomes prepared in the two media, the maximum fluorescence of the histograms correlates well with the loading data for **SRB** (higher in water than in W5E), **FITC4000** (distinctly higher in W5E than water), **FITC70000** (higher in water than W5E), **6‐FAM** (higher in W5E), **R6G** and **RhB** (higher in water) and **NB** (rather similar). **SfrO** and **RBP** show generally rather low intensities. Concerning the modality of the populations, single narrow populations are only found for porous cerasomes with **SRB**, **6‐FAM**, **NB** and **FITC70000** in water. **FITC4000** and **FITC70000** show broader populations in W5E. The same holds for **R6G** and especially **RhB**‐loaded cerasomes prepared in water, in the latter case possibly being connected to a higher heterogeneity of hydrophobic regions that are present in the porous compared with the non‐porous cerasomes.

### 2.5 pH Gating and Anion Sensing

For the loaded and gated cerasomes that could be obtained, a test of pore opening was performed next (Figure 7a,b, Figures S16 and S17). This was done by adding NaOH to cerasome suspensions of pH 3, to reach pH 9 and convert the brushes of protonated amines into linear gatekeeper molecules in their neutral state, allowing for the pH inside the void to equilibrate with the external one. Control experiments were carried out with the equivalent non‐porous loaded cerasomes in addition. For the cerasomes loaded with **SRB**, no conclusive observations could be made, because the absorption and emission spectra remained unchanged toward pH variations. Indeed, as the absorption and fluorescence properties of this dye are not sensitive to pH, equilibration of the medium inside of the cerasomes toward the surrounding solution would largely escape detection. For all the other systems, an immediate equilibration (< 10 s) was observed in contrast to the slow release from mesoporous materials (Figure S18d) [51].

**FIGURE 7:**
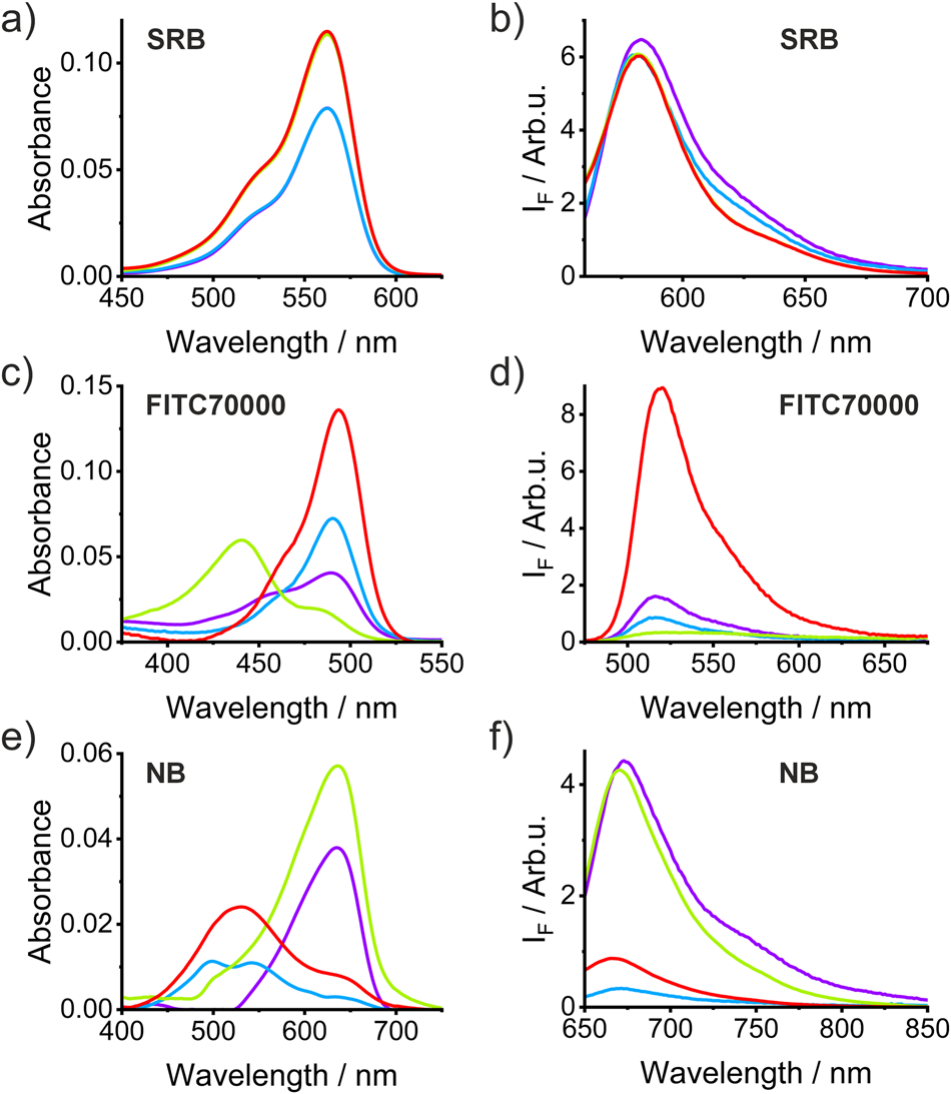
Absorption and corrected emission spectra of a solution containing porous cerasomes loaded with (a) and (b) **SRB**; (c) and (d) **FITC70000**; (e) and (f) **NB**) and functionalized with polyanion‐modulated pH‐responsive gates upon basification of the medium (purple line: non‐porous cerasomes at pH 3, blue line: non‐porous cerasomes at pH 9, green line: porous cerasomes at pH 3, red line: porous cerasomes at pH 9).

For **6‐FAM**, **FITC4000** and **FITC70000**, the control experiments indicated that the obtained non‐porous cerasomes might be slightly permeable to protons. The absorption bands of the dyes are moderately or strongly shifted due to a basic pH environment outside the cerasomes, but no drastic enhancement of the emission was observed, indicating that no complete equilibrium between the inside and the outside of the particles occurs [40, 41, 42]. For polyanion‐modulated pH‐gated cerasomes, a similar behaviour of the absorption spectra was observed, plus a strong emission enhancement of 6, 20 and 27‐fold for **6‐FAM**, **FITC4000** and **FITC70000**, respectively. Such behavior indicated that the pores were fully opened, and the pH in the cerasomes was rapidly equilibrated. Upon increasing the pH of solutions containing the **FITC70000** dye alone or the **FITC70000**‐loaded cerasomes (Figure S18), similar behavior was observed in absorption, typical as reported for fluorescein [33]. However, in emission, it can be clearly seen that the dye trapped inside the cerasomes is behaving differently. Even above the p*K*_a3_ = 6.4, where the highly emissive dianion starts dominating, as seen in absorption, and the emission should rise (as observed for **FITC70000** alone), no drastic increase is observed. This phenomenon could be explained by the inner‐filter effect and/or homo‐energy transfer with trapping‐related quenching within the cerasomes due to a high local concentration of fluorophores [54, 55], highlighting that the FITC‐dextran macromolecules remain trapped even upon opening of the pores. This was confirmed by performing a tentative SEC on basified **FITC70000**‐loaded cerasomes gated with **N3**, for which the **FITC70000** could no longer be separated. This reversed gating mechanism, where the indicator cargo remains inside the particles, yet the influx of species from the medium outside of the cerasomes into their inner void is of great interest regarding equilibrium kinetics and sensory response times. Indeed, gated systems based on mesoporous particles are almost always exhibiting long equilibration times [51], which necessitates the development of strategies for use in rapid analytical tools where equilibrium formation of the system components may be inherently slow [10]. For **R6G**, the control experiment showed no response, while for gated cerasomes, a strong quenching of the dye emission was observed at basic pH, similar to **RhB** [56].

To further test the controlled pore opening capacity of this type of particulate object, the gated cerasomes containing **FITC70000**, which are giving the strongest response upon opening of the pores (27‐fold) by conversion of the fluorescein moieties, were selected for adenosine 5′‐triphosphate (ATP) sensing as a model application. Indeed, cerasomes would be an ideal platform for biosensors, presenting low toxicity and high robustness while being able to penetrate cells [27, 44]. Based on previous studies [51], the polyanion‐modulated pH‐gating mechanism would be hindered in the presence of big anions such as adenosine triphosphate (ATP) through electrostatic interaction of the anion and the partially protonated amino groups of the polyamines grafted to the surface (Figure 8a), allowing for ATP detection. For this purpose, cerasome suspensions (at pH 3) were added to solutions containing increasing concentrations of ATP from 0 to 10 mm in Milli‐Q water at pH 7 (Cerasome/Sample 2.5/100 v/v), the addition of a concentrated cerasome solution even at pH 3 ensuring that the volume and pH of the final solution were minimally affected. The system response upon conversion of the FITC labels of the **FITC70000** into their dianionic form was found to be inversely proportional to the ATP concentration (Figure S19), confirming that big and multivalent anions like ATP can prevent the opening of the **N3** gate and equilibration of the pH within the cerasome void. The zeta potential analysis (Figure S19e) showed that the cerasomes’ surface remained positively charged until addition of more than 10 µm of ATP, but for higher concentrations the value turned negative due to surface saturation and ATP buffer effect as detailed below. The concentration of **FITC70000** converted from neutral to dianionic form and vice versa by the change of 1 order of magnitude of ATP was estimated to approximately 0.6 µm on average. But for concentrations of ATP higher than 10 µm an important decrease of the pH was observed (Figure S20), and system response was dually affected by the increasing ATP concentration and the lowering of pH. Using water buffered at pH 7.3 (10 mm HEPES‐NaOH) enabled stable pH conditions up to an ATP concentration of 1 mm (Figure S20). Under these conditions, the dianionic form of **FITC70000** was predominantly present within the cerasome void (Figure S21b). The absorption response remained essentially unchanged (Figure S21a), whereas the fluorescence emission of **FITC70000** increased together with the ATP concentration following a sigmoidal trend (Figure 8b,c). The observed behavior may be related to the influence of HEPES on proton‐exchange dynamics. Sulfonate‐containing buffers such as HEPES have been reported to accelerate proton‐transfer processes involving fluorescein and related fluorophores, thereby affecting protonation equilibria and exchange kinetics of the dye [57]. At low ATP concentrations, the presence of buffer species within the cerasome interior may alter the local protonation dynamics of **FITC70000**, thereby influencing the observed fluorescence intensity. As the ATP concentration increases, occupation of the cerasome surface by ATP is expected to reduce the accessibility of buffer molecules to the confined fluorophores, resulting in enhanced fluorescence emission. Noteworthy, when the buffering capacity of the system is exceeded and ATP‐induced acidification again becomes dominant, pronounced fluorescence quenching was observed as in the absence of buffer. From the measured spectra, a calibration plot was constructed, allowing to determine a limit of detection of 0.02 µm (relative uncertainty method [58, 59]) with a broad dynamic range up to 1 mm.

**FIGURE 8:**
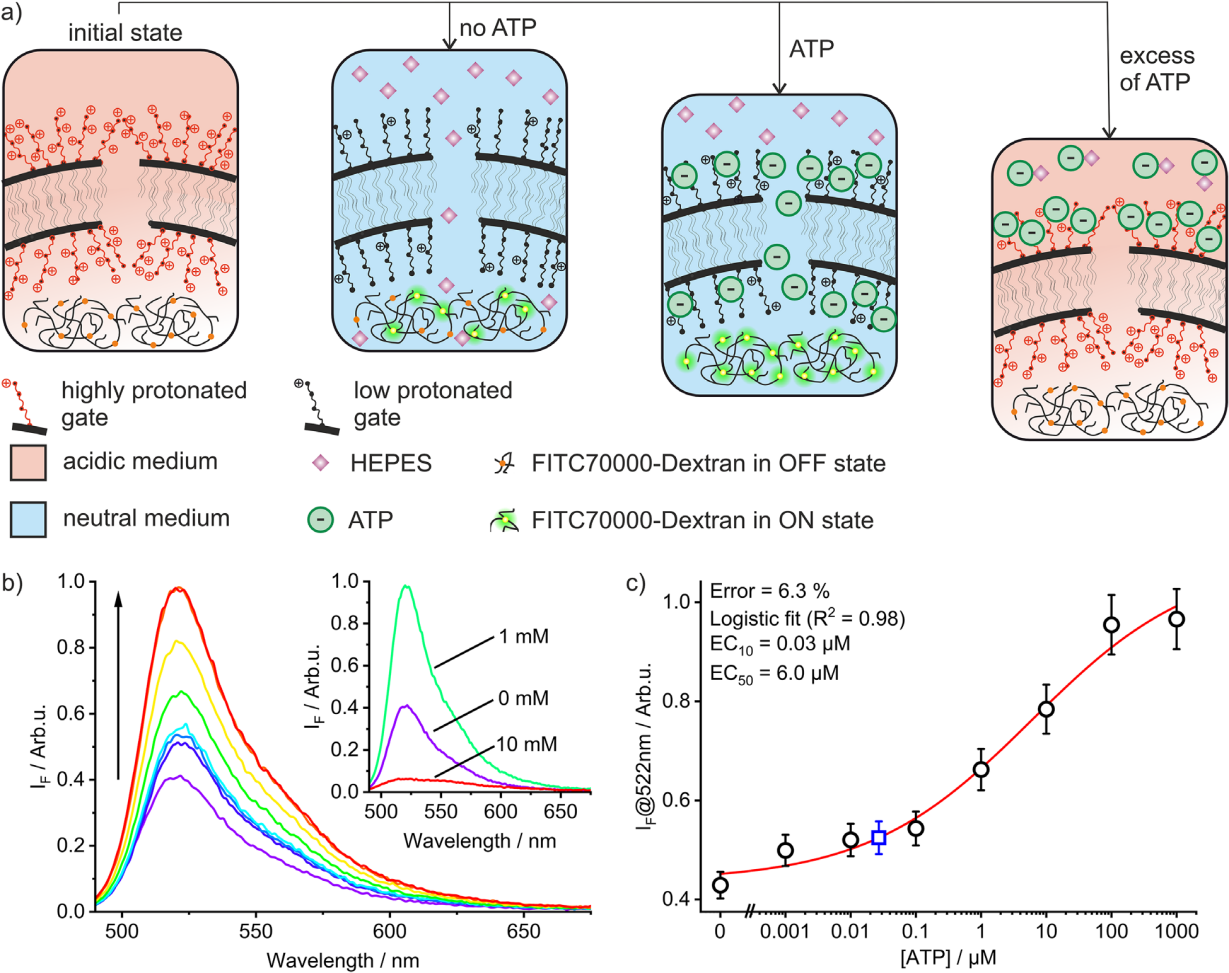
(a) Scheme of the gated material in the initial state (as stored) in acidic medium and upon addition to neutral medium (HEPES‐NaOH, 10 mM, pH 7.3) in absence, presence and excess of ATP allowing or preventing the opening of the pores and ionic strength equilibration and inducing pH drop (in case of ATP excess); (b) Emission spectra of a solution containing **FITC70000**‐loaded porous cerasomes with pH/anion responsive gates upon increasing concentration of ATP (inset with response upon excess of ATP (10 mm) inducing a pH drop); (c) Calibration plot of sensory material response for ATP, blue square is corresponding to the response of the swab test of a contaminated surface (HEPES‐NaOH at 10 mm – pH 7.3; *λ*_exc_ = 480 nm).

The selectivity of the sensing system toward competing anions was investigated (Figure S21c). Given the nature of the gating mechanism, high analyte selectivity was not anticipated, as anions possessing similar p*K*_a_ to ATP may interact with the gate in a comparable manner. Indeed, citrate induced partial pore blocking, whereas ADP, AMP, glycine and phosphate promoted partial pore opening. However, subsequent addition of 10 µm ATP produced a response equivalent to that observed in the absence of competing species, demonstrating that the sensor mostly retained its functionality under these conditions.

To demonstrate the practical applicability of the sensing system for hygiene monitoring, a dry saliva‐contaminated glass surface was tested (Figure S22). In brief, a sterile swab was pre‐wetted, used to swipe the surface and then immersed in HEPES‐NaOH buffer to extract the analytes collected. The cerasome suspension was then added, and the resulting fluorescence response was recorded. The swab test produced a positive response in the presence of dried saliva residues. Based on the calibration curve established above, the recovered ATP amount was estimated to be 40 ± 32 pmol from the sampled surface. In contrast, a clean glass surface, without saliva contamination, produced no detectable response.

In comparison to recently reported sensors for ATP (Table S2), the developed system shows excellent sensitivity, a broad dynamic range and a fast response time.

## 3 Conclusions

In this work, we prepared nanohybrid materials that are analogous to liposomes but stabilized by two ultrathin silica monolayers, called cerasomes, according to previous reports with an improved protocol for the synthesis of the precursor lipid. Being interested in gated functions of dye‐loaded nanoscopic objects, we investigated the potential of loading a palette of fluorescent dyes differing in size, permanent or pH‐dependent charge state and hydrophilicity into the cerasomes, comparing non‐porous and porous cerasomes. For the latter, we successfully adapted protocols from liposome and mesoporous silica chemistry, yielding cerasomes with porous membranes which were subsequently loaded with dyes and equipped with a pH‐responsive gating chemistry. All particulate objects were characterized with various techniques, including SEM imaging. Especially the ultrathin silica surfaces allowed for a facile grafting of functional entities via sol–gel techniques, using linear polyamine silanes here but also being expandable to attaching haptens for immunoassays or antifouling entities.

The in‐depth dye loading and pH‐switching studies revealed that porous cerasomes loaded with **FITC70000**, a commercial dextran that is labelled with a considerably small number of fluorescein dyes (FITC), perform best in terms of pH‐controlled fluorescence changes. This system allowed us to prove that porous cerasomes operate different to other gating systems based on mesoporous silica particles, i.e., that not a gated release of the dyes contained in the cerasomes is observed, but that the influx of the chemical stimuli itself from the surrounding medium into the liposome‐like objects occurs. The advantage of this reversed gating mechanism is the response time, which is much faster (<10 s) than for conventional gated release systems (5 min to several hours). This approach was successfully validated with an assay for the detection of ATP as a model anion, the target analyte modulating the pH gating of the **FITC70000**‐loaded cerasomes carrying polyaminic gatekeepers on its surface. The obtained results were coherent with previous studies, and concentrations from 0.02 µmm to 1 mm could be detected. Such fluorescent particle probes with low detection limit and wide dynamic range could, for instance, be advantageously integrated into swab testing methods for surface analyses as demonstrated [60, 61, 62]. Current ongoing studies are concerned with the optimization of the materials preparation for specific applications, i.e., alternatives to poloxamers will be screened to obtain more uniform pores of controlled size, the grafting of alternative molecular gates for the realisation of bioassays will be tested, and the integration of such systems into sensory platforms such as lateral flow assays or microfluidic devices. While the dye‐labelled macromolecule **FITC70000** performed best with the gated porous cerasomes, especially the experiments with the non‐porous cerasomes and **NB** were intriguing as they suggested that also the membranes of these cerasomes are permeable to protons, potentially allowing the use a wealth of dyes in their molecular form together with non‐porous cerasomes and an outer gating chemistry for stimuli‐responsive purposes.

## Author Contributions

**R. Vinot**: Investigation, Methodology, Validation, Writing – review & Editing. **E. Climent**: Conceptualization, Funding acquisition, Supervision, Writing – Review & Editing. **M. Friedrich**: Investigation, Writing – Review & Editing. **K. Rurack**: Conceptualization, Formal analysis, Investigation, Resources, Supervision, Writing – Review & Editing. **J. Bell**: Conceptualization, Data curation, Formal analysis, Investigation, Methodology, Supervision, Validation, Visualization, Writing – Original Draft, Writing – Review & Editing.

## Funding

This research was supported by the German Research Foundation (DFG; CL 761/1‐19)

## Conflicts of Interest

The authors declare no conflicts of interest.

## Supporting information




**Supporting File**: smll75507‐sup‐0001‐SuppMat.pdf.

## Data Availability

The authors declare that the data supporting the findings of this study are available within the paper and its Supplementary Information files. Should any raw data files be needed in another format, they are available from the corresponding author upon reasonable request.
