# Supplementary material for: Fluorescence Sensing of Anions Like Adenosine 5ʹ‐Triphosphate With pH‐Gated Porous Dye‐Loaded Cerasomes
Source: Small. 2026 Aug 31;22(61):e75507. doi: 10.1002/smll.75507 (PMC13725399; doi:10.1002/smll.75507)
Supplement: Supplementary file 1 [file SMLL-22-e75507-s001.pdf]

Supporting Information

Fluorescence sensing of anions like adenosine 5′-triphosphate with pH-gated porous dye-loaded cerasomes

Romane Vinot, Estela Climent, Maja Friedrich, Jérémy Bell and Knut Rurack*

Chemical and Optical Sensing Division, Bundesanstalt für Materialforschung und -prüfung (BAM), Richard-Willstätter-Str. 11, 12489 Berlin, Germany.

E-mail: jeremy.bell@bam.de

Table of content

I	Material and methods	S2
II	Synthesis of the cerasome precursor	S3
III	Preparation and characterisation of non-porous cerasomes	S5
IV	Preparation and characterisation of porous cerasomes	S15
V	Response to ATP	S20
VI	References	S24

I. Material and methods

All chemicals were purchased from Sigma-Aldrich, ABCR or Applichem and used without further purification. All organic solvents were of HPLC grade quality and used without further purification. Water was drawn from a Milli-Q ultrapure water purification system (Millipore Synthesis A10). For the experiments carried out in water with 5% ethanol, the solvent mixture is abbreviated W5E. For column chromatography, silica gel (0.035–0.070 mm, 60 Å) was used. Size exclusion column chromatography was realized with Prepacked Disposable PD-10 columns from GE Healthcare. NMR spectra were recorded on a Varian Mercury 400 NMR spectrometer. The hydrodynamic diameter and polydispersity index of the cerasomes were measured by dynamic light scattering together with the Zeta potential on a Zetasizer Nano-ZS from Malvern. Flow cytometric analyses were performed on a BD Accuri C6 or a CyFlow space from Partec (488 nm excitation, 520/590 nm emission collection). High-resolution scanning electron microscopy (SEM) was performed on a Zeiss Supra 40 equipped with a high-resolution cathode (Schottky field emitter) with an In-Lens SE secondary electron detector.

The formation of the cerasomes was followed by flow cytometry, DLS and zeta potential measurements. On one hand, flow cytometry and DLS through light scattering measurements allowed us to monitor the size and morphology changes as a function of time. On the other hand, changes of the zeta potential due to the crosslinking of the silica shell at the cerasomes surface allowed to monitor the formation of the cerasome membrane. Moreover, loading of the cerasomes and opening of the pores was followed by flow cytometry and conventional spectroscopic methods. As a comparison, Scanning Electron Microscopy measurements allowed us to corroborate the formation of cerasomes.

II. Synthesis of the cerasome precursor

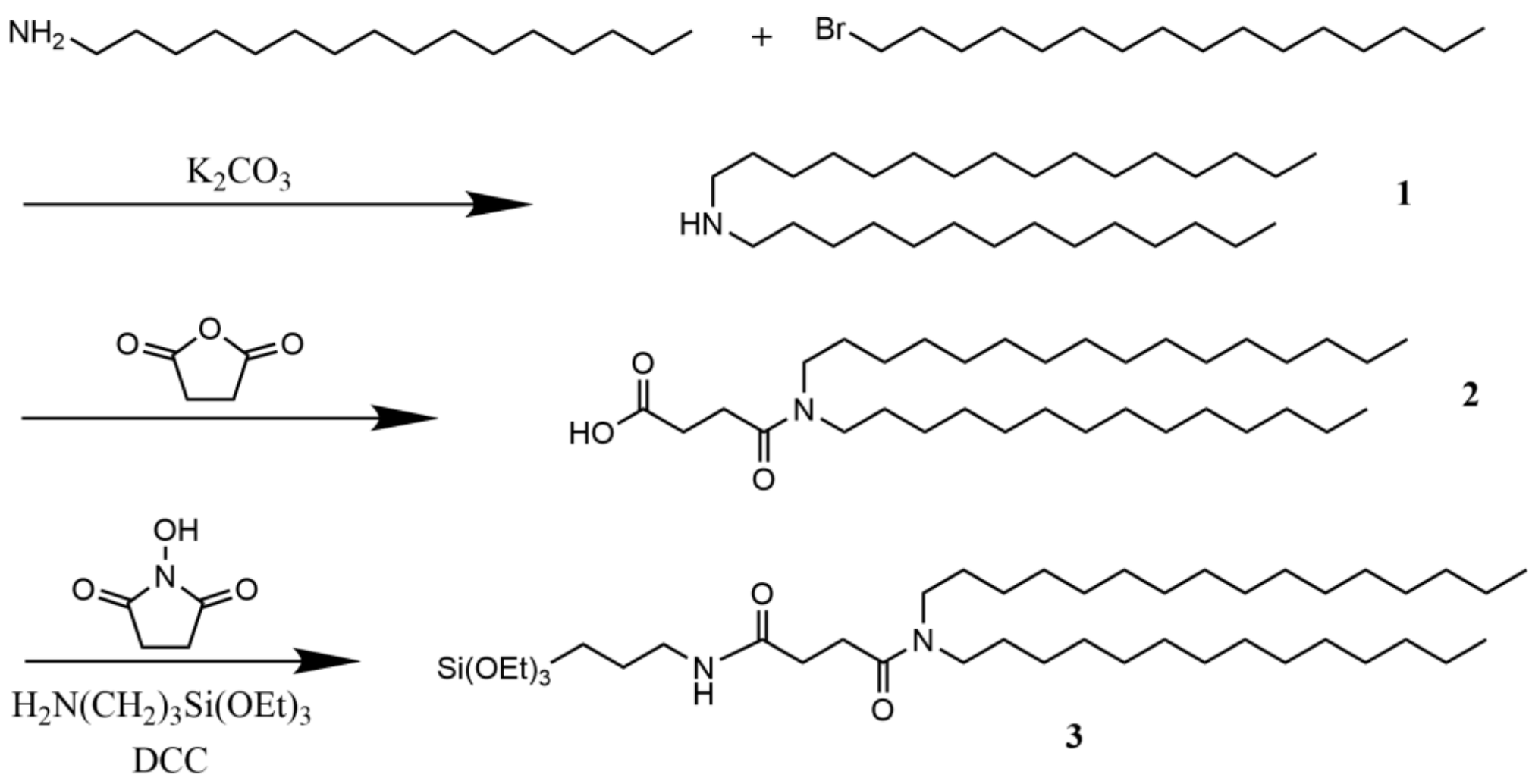

Figure S1. Scheme of the synthetic route to the precursor lipid **3**

N,*N*-Dihexadecylamine **1** and *N*,*N*-dihexadecylsuccinamic acid **2** were prepared as reported previously [1].

N-[*N*-(3-Triethoxysilyl)propylsuccinamoyl]dihexadecylamine **3**: 1.26 g (6.1 mmol, 1.5 equiv.) of *N*,*N'*-dicyclohexylcarbodiimide (DCC) and 0.51 g (4.5 mmol, 1.1 equiv.) of *N*-hydroxysuccinimide (NHS) were added to a solution of *N*,*N*-dihexadecylsuccinamic acid (2.5 g, 4.4 mmol) in dry dioxane (100 mL) under argon while stirring at room temperature. After 5h stirring at RT, the mixture was centrifuged and the urea (*N*,*N'*-dicyclohexylurea) already formed removed by centrifugation and filtration. 1.14 mL (4.9 mmol, 1.2 equiv.) of 3-Aminopropyltriethoxysilane was added to the solution and the mixture was stirred for 16 h at RT. Precipitates (*N*,*N'*-dicyclohexylurea) were removed by filtration. The solvent was evaporated under vacuum giving a colourless oil. Chromatographic purification on a non-pre-packed and short (4 cm) silica column was realized. First, impurities were eluted with ethyl acetate/chloroform 1:9; a subsequent elution with 100% ethyl acetate afforded the pure lipid **3** (yield 77%). ^{1}H NMR (400 MHz, $CDCl_3$, , TMS): d= 0.62 (t, *J*=8.4 Hz, 2H, $SiCH_2$), 0.88 (t, *J*=6.6 Hz, 6H), 1.22 (t, *J*=7.3 Hz, 9H), 1.22–1.29 (m, 52H), 1.60 (br, 6H), 2.51 (t, *J*=6.6 Hz, 2H), 2.64 (t, *J*=6.6 Hz, 2H), 3.15–3.24 (m, 6H), 3.80 (q, *J*=7.3 Hz, 6H), 6.30 ppm (br, 1H).

Lipid **3** was subsequently dissolved in ethanol and divided into several fractions of 3 mg of precursor lipid **3**. Ethanol was evaporated with argon, and the fractions were stored in a freezer at –20°C.

III. Preparation and characterisation of non-porous cerasomes

Preparation of non-porous cerasomes

A fraction of 3 mg of lipid **3** was thawed and dissolved in 300 µL of ethanol. This solution was divided in 6 aliquots, and then ethanol was evaporated with argon to have a thin layer of lipid on the Eppendorf vial's wall. The lipid was dissolved in 1 mL of Milli-Q water at pH 3 (HCl), optimal pH for the formation of the silanol network. This solution was mixed with a vortex mixer for 10 min and subsequently sonicated for 5 min (75% amplitude, cycle 50%) with a sonotrode (Hielscher UP200S) equipped with a S1 probe (1 mm, titanium) inducing a temperature rise between 50 °C and 60 °C for a power of approximately 75 W. The solution was left in the refrigerator for some days to let the formation of cerasomes occur (between 2 and 7 days). If after one week, cerasomes were still not forming, the solution was again mixed at room temperature for 12 h to help finishing the formation of the silanol network. Once the formation of cerasomes was finished, the solution was basified to obtain a pH between 5 and 7. An additional size exclusion chromatography (SEC) with water was performed to remove micelles and precursor lipid that did not react.

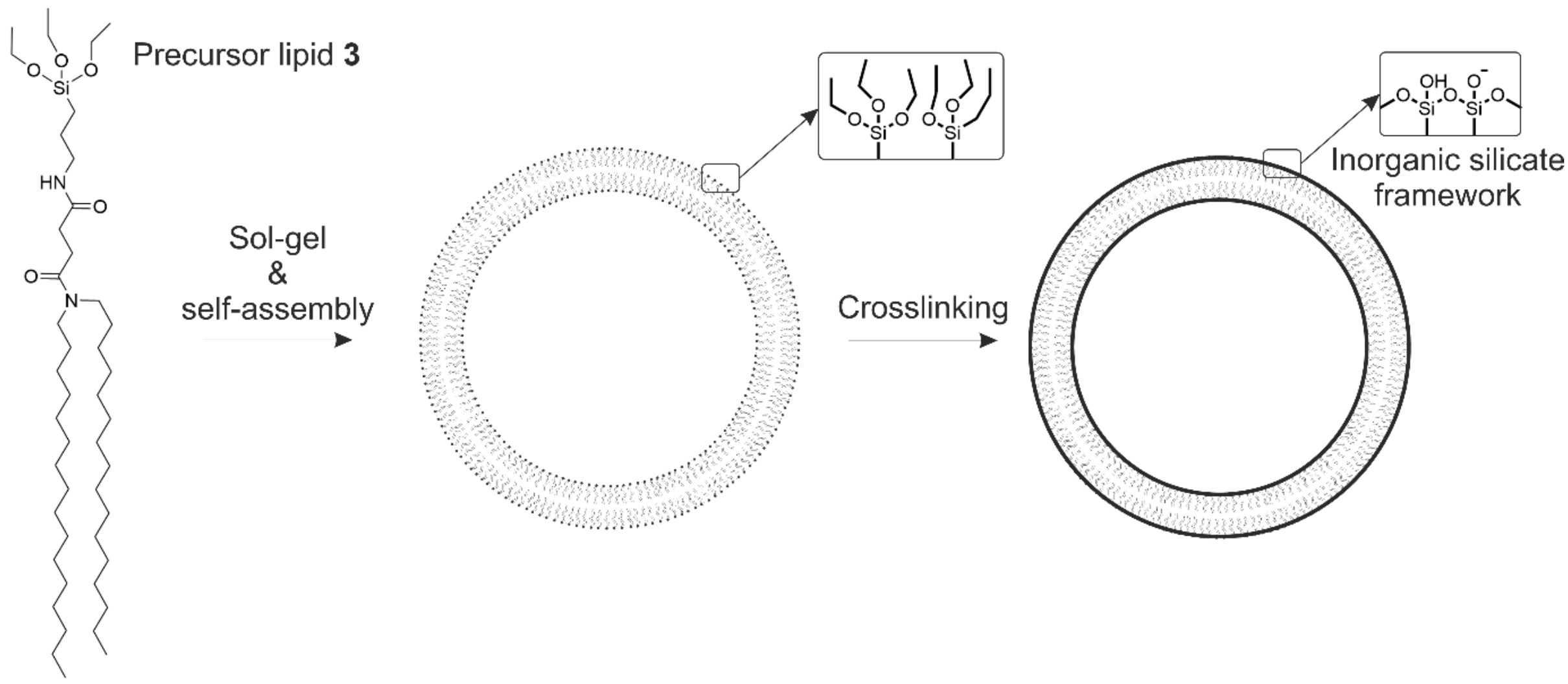

Figure S2. Self-assembly of the precursor lipid into micelles from a sol-gel mixture and progressive crosslinking of the silanol heads

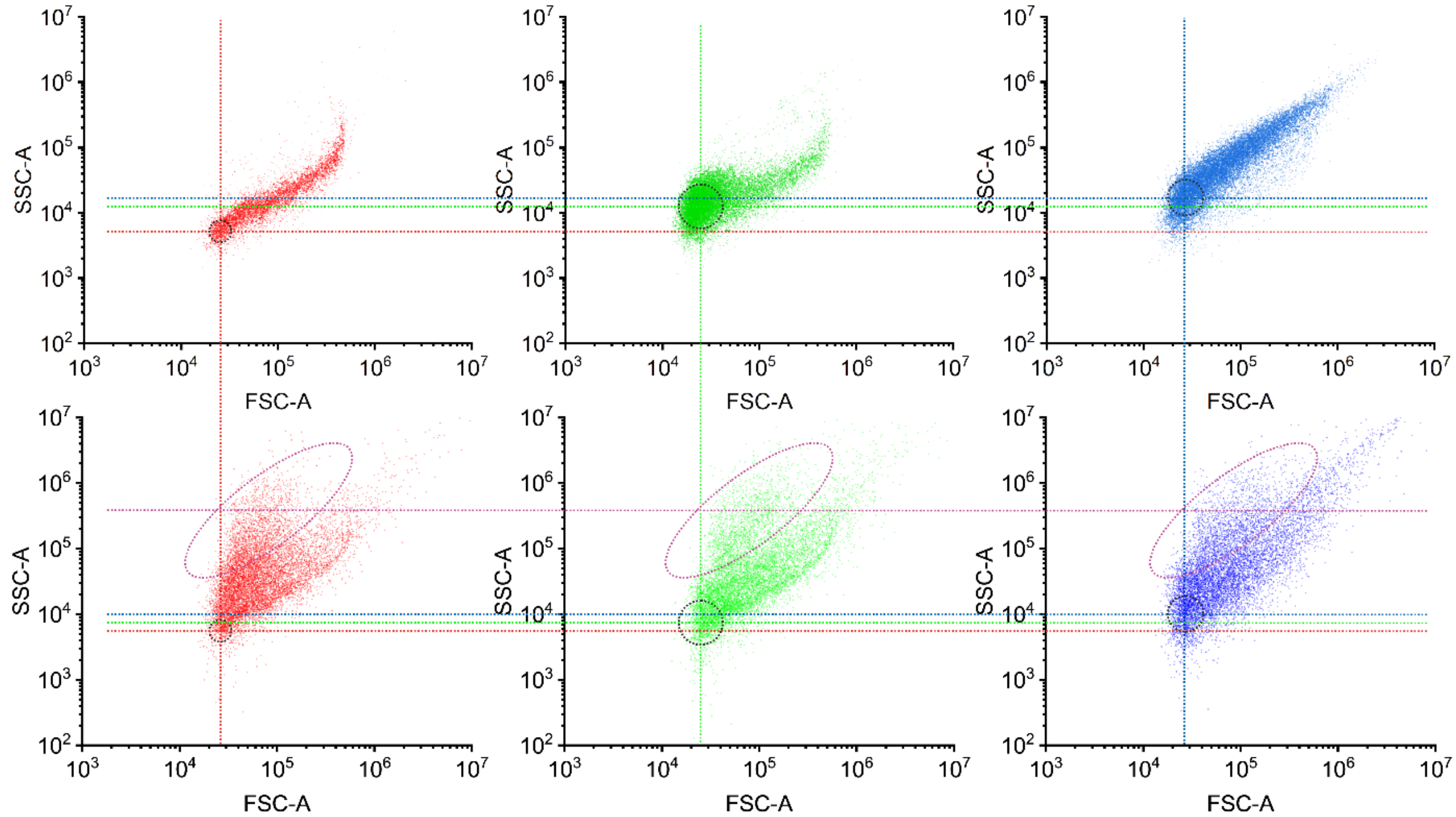

Figure S3. Different stages of cerasome synthesis monitored with a flow cytometer (non-porous top; porous bottom; reaction time increases from left to right): To monitor the progressive crosslinking of the micelles to form cerasomes, undiluted aliquots were taken from the reaction solution and directly injected into the cytometer. For such an analysis, the objects themselves are too small, and the object concentration is too high for single object detection events to occur so that these traces resemble “cerasome swarms”, i.e., multi-object detection events that lead to the characteristic “comet tail” traces. Important for synthesis control is thus the signal increase in the side scatter channel (SSC) with virtually unchanged signals in the forward scatter channel (FSC), showing that primarily the granularity of the objects (the major feature detected by SSC) is changing during the crosslinking of the micelles to form the cerasomes and not the size (the major feature detected by FSC). The coloured dotted lines and black dotted circles resemble the midpoints in FSC/SSC for the onset of the comet tail and are only guides to the eye, helping to illustrate how FSC stays rather constant along the synthesis, but SSC increases progressively. For the porous cerasomes, the pink dotted ovoid and line are highlighting the presence of poloxamer micelles stable during the whole cerasome process formation.

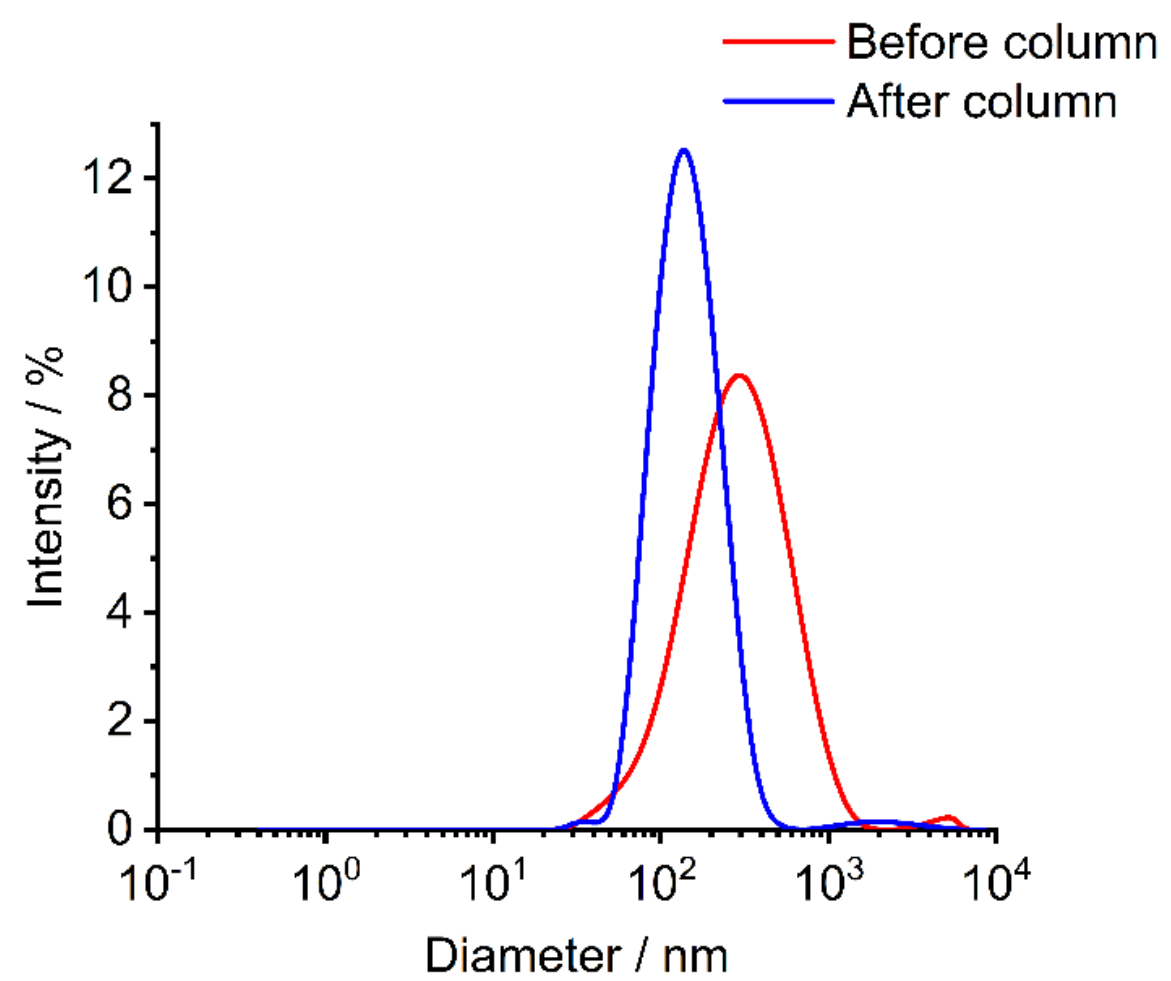

Figure S4. Typical graph of size measurements with DLS (hydrodynamic diameter) obtained after basification of the non-porous cerasome suspension before (red) and after (blue) size exclusion chromatography.

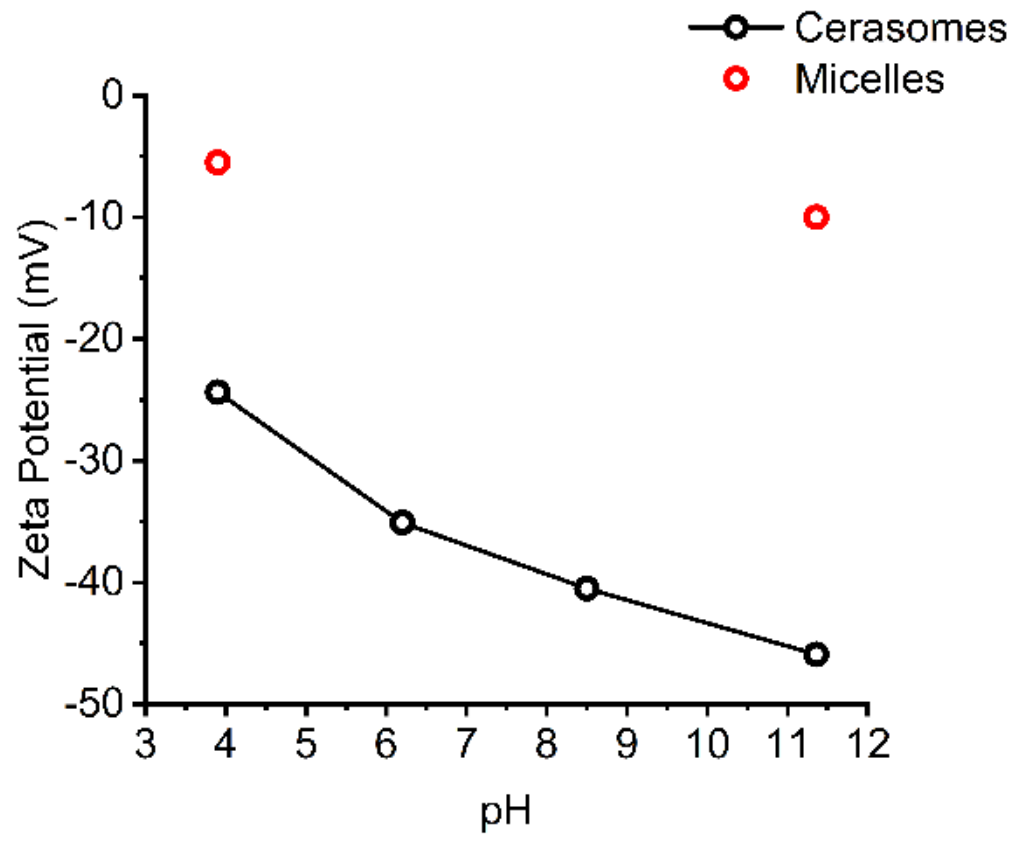

Figure S5. Zeta potential of micelles and cerasome suspensions in Milli-Q water as a function of the pH

Preparation of dye-loaded non-porous cerasomes through co-encapsulation

Cerasomes were also synthesised in the presence of dyes; rhodamine 6G chloride (CAS Number: 989-38-8) (**R6G**), rhodamine B chloride (CAS Number: 81-88-9) (**RhB**), sulforhodamine B sodium salt (CAS Number: 3520-42-1) (**SRB**), safranin O chloride (CAS Number: 477-73-6) (**SfrO**), tris(bipyridine)ruthenium(II) chloride (CAS Number: 50525-27-4) (**RBP**), nile blue chloride (CAS Number: 2381-85-3) (**NB**), 6-carboxyfluorescein (CAS Number: 3301-79-9) (**6-FAM**) and two different dye-labelled polymers (FITC-dextran, CAS Number: 60842-46-8, both with FITC : glucose = 0.008) **FITC4000** (MW ≈ 4000 g mol^{-1}) and **FITC70000** (MW ≈ 70000 g mol^{-1}) for encapsulation. These cerasomes were obtained with the same protocol described above with the addition of 300 µL of a dye solution (4 g L^{-1}, except for **FITC70000** with 40 g L^{-1} at pH 3 in water) and employing an additional SEC step with water to clean the cerasomes from micelles, precursor lipid that did not react and excess of dye.

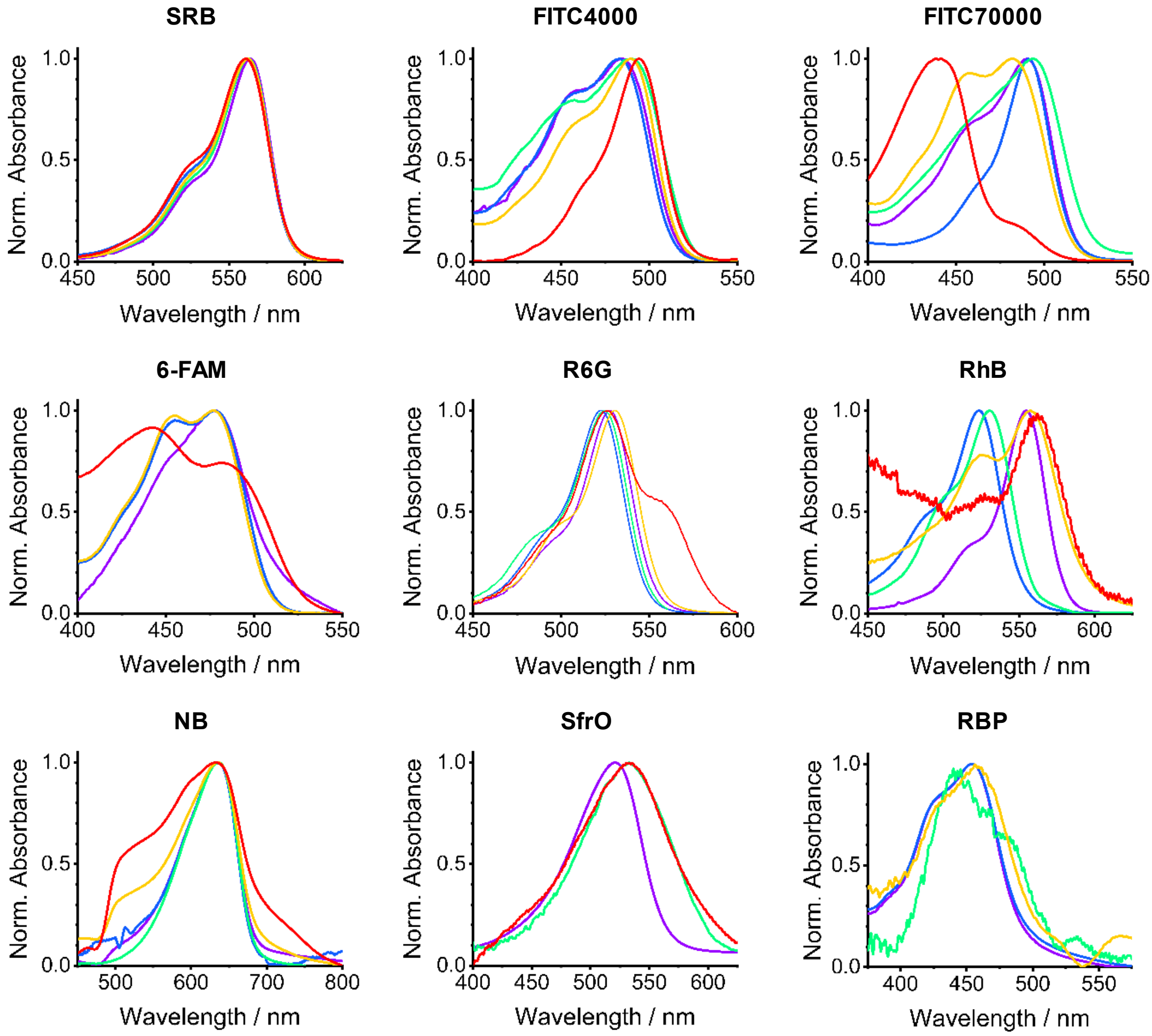

Figure S6. Normalized absorption spectra of the dyes (1 μM) and loaded cerasomes (purple lines: dyes in solution; blue lines: non-porous cerasomes formed and loaded in pure Milli-Q water; green lines: porous cerasomes loaded in pure Milli-Q water, yellow lines: non-porous cerasomes formed and loaded in Milli-Q water – ethanol 95/5 v/v mixture; red lines: porous cerasomes loaded in Milli-Q water – ethanol 95/5 v/v mixture). Due to non-negligible shift or dual bands observed for some of the dyes, the integral of the absorption bands for all dyes were used to calculate the dye concentration in cerasome suspension after synthesis and cleanup in Table S1.

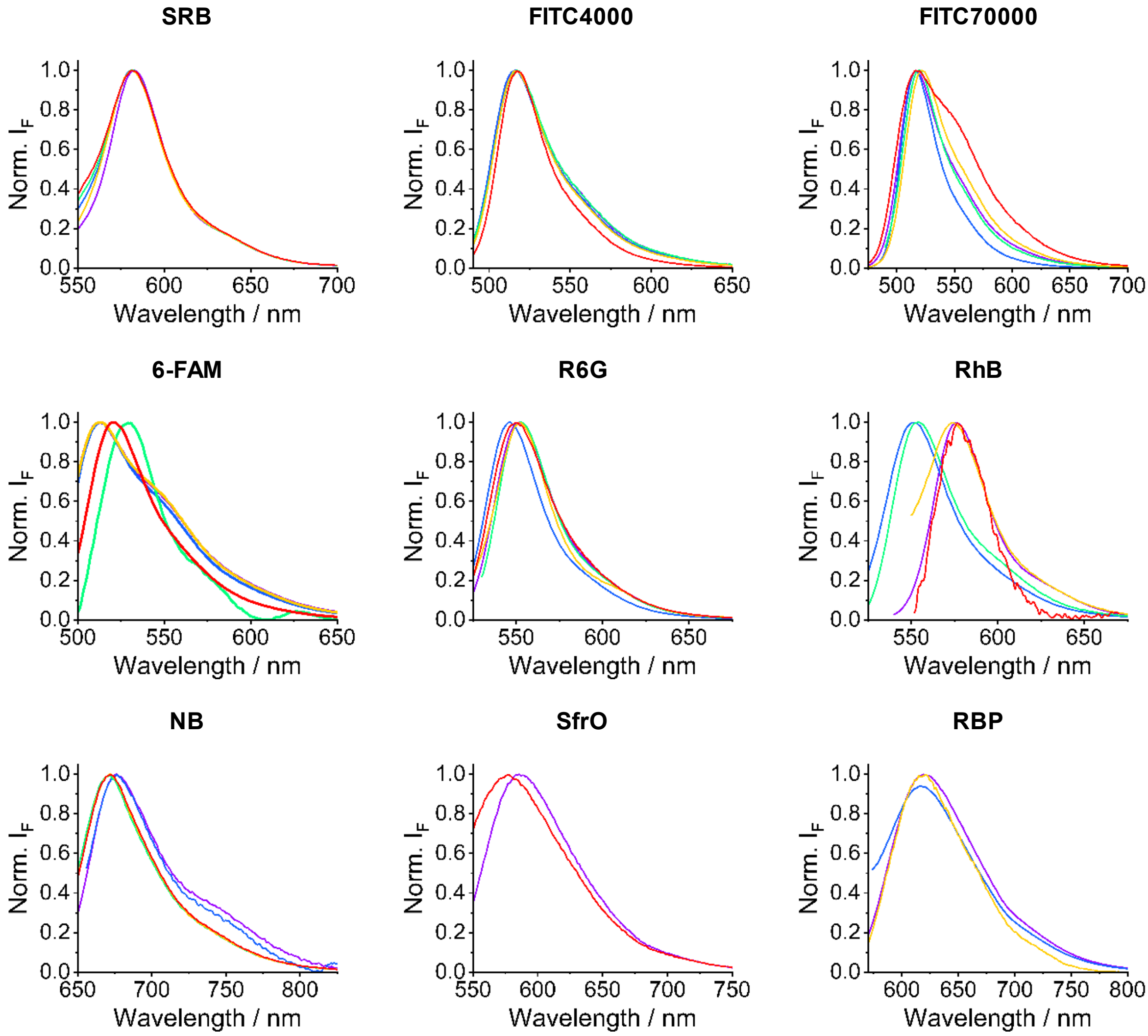

Figure S7. Normalized emission spectra of the dyes (1 μM) and encapsulated/loaded cerasomes (purple lines: dye solution; blue lines: non-porous cerasomes formed and loaded in pure Milli-Q water; green lines: porous cerasomes loaded in pure Milli-Q water, yellow lines: non-porous cerasomes formed and loaded in Milli-Q water – ethanol 95/5 v/v mixture (W5E); red lines: porous cerasomes loaded in Milli-Q water – ethanol 95/5 v/v mixture (W5E)).

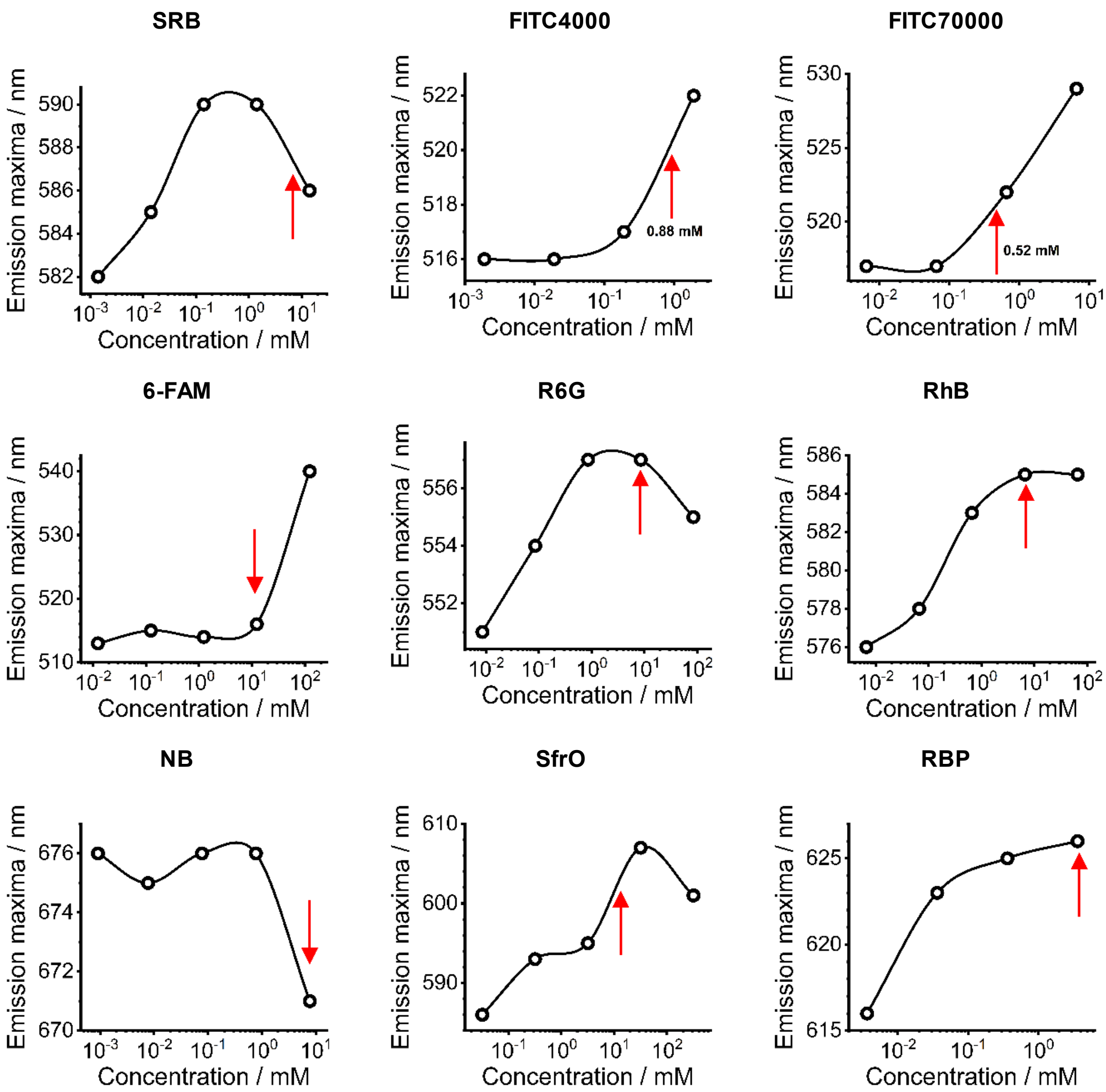

Figure S8. Dependency of the dyes' emission maxima *vs* their concentration in water; measurements in front face setup; Red arrows indicate the concentration used for encapsulation or loading into the cerasomes.

Table S1. Approximation of the encapsulation efficiencies EE_{abs} into non-porous cerasomes and loading efficiencies LE_{abs} into porous cerasomes of the dyes as derived from spectroscopic measurements. The concentrations of the dyes were determined via absorption measurements and molar absorption coefficients extracted from band integrals ε_{int} according to the Beer-Lambert law.

Dye	$\varepsilon_{\lambda_{max}}$ /M^{-1} cm^{-1}	ε_{int} /10^6 M^{-1} cm^{-1}	Φ_f	% EtOH	c_{dye}^{init} [a] /mM	Non-porous cerasomes			Porous cerasomes		
						c_{dye}^{susp} [b] /µM	λ_{max}^{em} /nm	EE_{abs} [c] %	c_{dye}^{susp} [b] /µM	λ_{max}^{em} /nm	LE_{abs} [c] %
SRB	95950 [2]	4.43	0.9 [3]	0	8.4	7.6	581	0.1	67.6	581	0.8
FITC4000	38050	2.77	n.d.[d]	0	0.9	1.6	516	0.2	2.2	517	0.3
FITC70000	53250	4.91	n.d.	0	0.5	1.5	517	0.3	64.3	519	12.4
6FAM	76900 [4]	8.62	0.9 [3]	0	10.6	8.8	513	0.1	16.8	516	0.2
R6G	87000 [5]	3.78	0.9 [3]	0	8.6	1.9	546	<0.1	33.0	553	0.4
RhB	97520 [2]	4.47	0.95 [3]	0	8.8	3.0	551	<0.1	3.0	552	<0.1
NB	33000 [6]	5.47	0.01 [7]	0	12.6	2.5	676	<0.1	324.0	672	2.6
SfrO	31200 [8]	3.15	0.06 [8]	0	11.4	*no encapsulation*			*no loaded particles*		
RBP	14500 [9]	1.00	0.8 [3]	0	6.1	34.7	621	0.6	*no loaded particles*		
SRB	n.d.	n.d.	n.d.	5	8.4	37.4	582	0.4	1.7	582	<0.1
FITC4000	n.d.	n.d.	n.d.	5	0.9	14.9	517	1.7	32.8	517	3.7
FITC70000	n.d.	n.d.	n.d.	5	0.5	82.2	517	15.9	31.1	517	6.0
6FAM	n.d.	n.d.	n.d.	5	10.6	636.0	514	6.0	23.9	520	0.2
R6G	n.d.	n.d.	n.d.	5	8.6	0.3	552	<0.1	0.3	550	<0.1
RhB	n.d.	n.d.	n.d.	5	8.8	0.3	574	<0.1	<0.1	577	<0.1
NB	n.d.	n.d.	n.d.	5	12.6	33.1	673	0.3	185.0	672	1.5
SfrO	n.d.	n.d.	n.d.	5	11.4	*no encapsulation*			0.6	577	<0.1
RBP	n.d.	n.d.	n.d.	5	6.1	1.2	617	<0.1	*no loaded particles*		

[a] Initial dye concentration used in synthesis. [b] Dye concentration in cerasome suspension after synthesis and cleanup. [c] Encapsulation efficiency (EE_{abs}) and Loading efficiency (LE_{abs}) obtained from the quotient of incorporated dye concentration and the initial dye concentration used in the synthesis. [d] Not determined or reported.

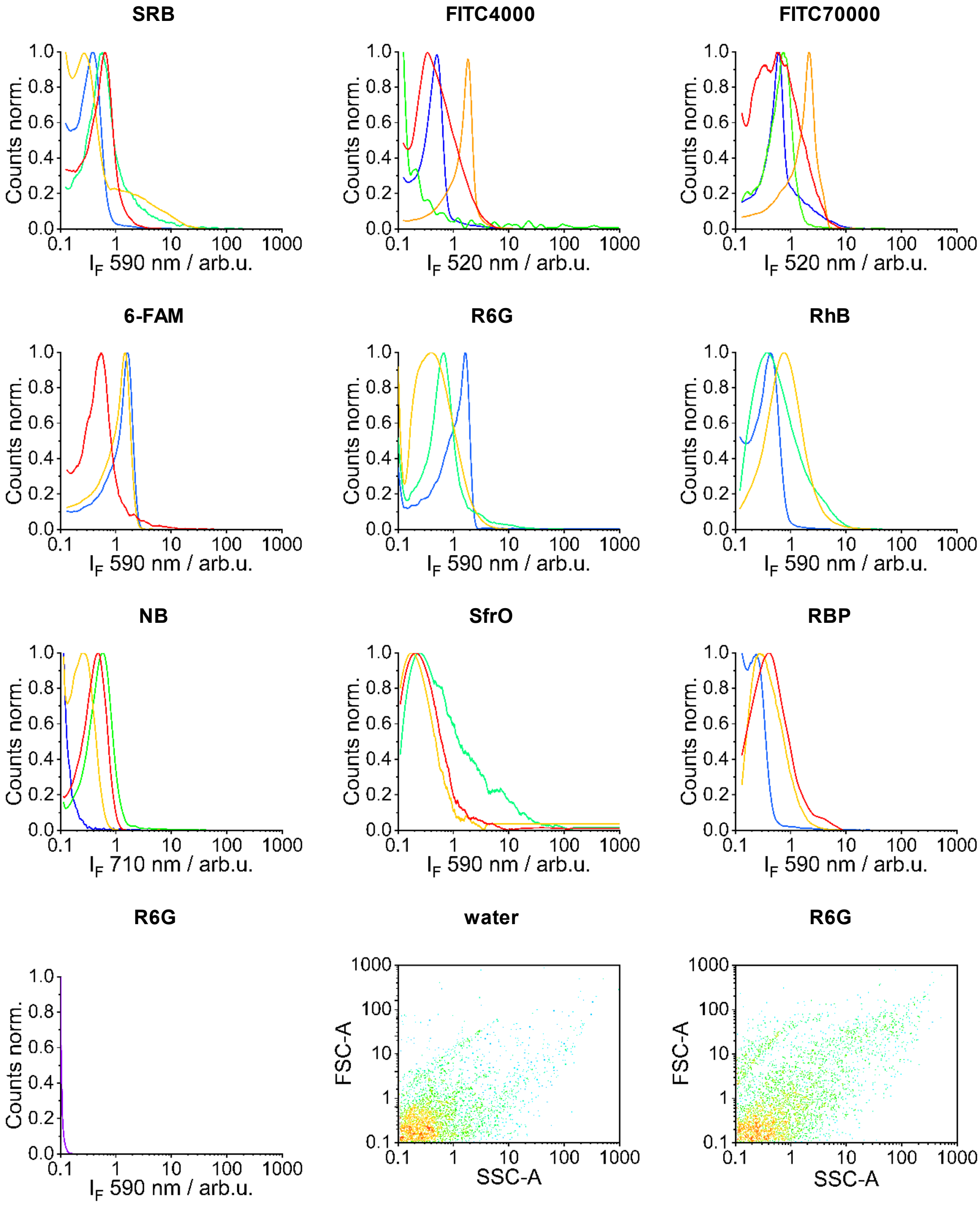

Figure S9. Flow cytometry emission histograms obtained for the dye-loaded cerasomes: blue lines–non-porous cerasomes formed and loaded in Milli-Q water; green lines–porous cerasomes loaded in Milli-Q water; yellow lines–non-porous cerasomes formed and loaded in W5E; red lines–porous cerasomes loaded in W5E. The last row contains a representative histogram of **R6G** in water (results for W5E were similar) and two representative dot plots for water (i.e., background; results for W5E were similar) and diluted cerasomes (**R6G** encapsulated in non-porous cerasomes in water/W5E). No gating was applied for the histograms.

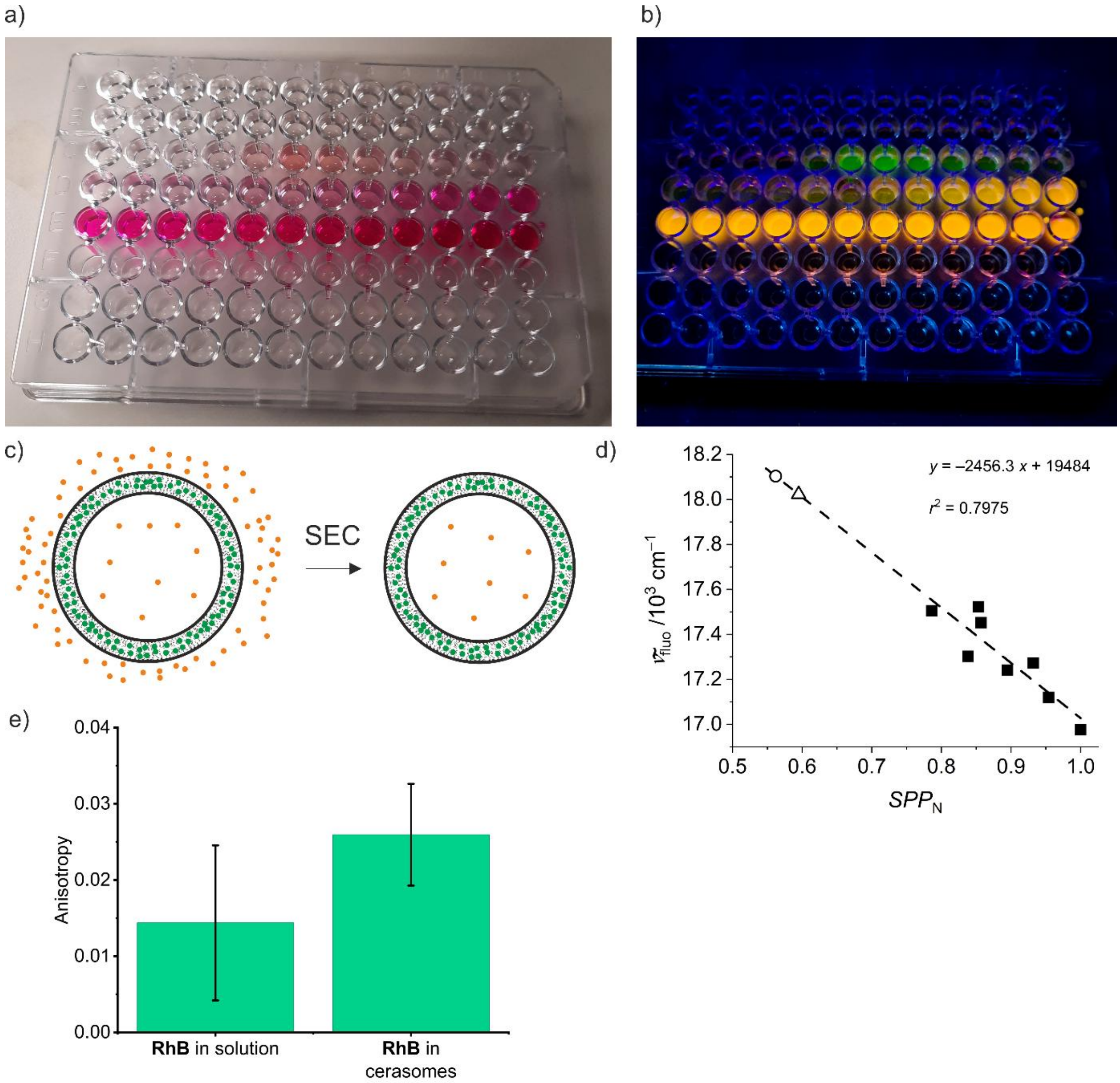

Figure S10. a) and b) Photographs under day and UV light of the microwell plate containing the washing fractions from SEC with **RhB** encapsulated non-porous cerasomes (greenish fluorescence) and free **RhB** (reddish fluorescence) (λ_{exc} = 365 nm); c) Tentative encapsulation scenario for **RhB** in the hydrophobic lipid interlayer of the non-porous cerasomes; states before and after size exclusion chromatographic cleanup; d) Correlation between fluorescence emission maximum of **RhB** in various solvents and the empirical solvent dipolarity and polarizability scale SPP_N (■) [10]; the solvents in increasing SPP_N order are $CHCl_3$, THF, EtOH, MeOH, MeCN, ethylene glycol, DMF and DMSO; fit parameters indicated in the plot; the instrumentally corrected emission spectra have been further corrected after conversion to the wavenumber scale [11]; ○ and △ correspond to emission maxima of **RhB** in non-porous (○, SPP_N = 0.562) and porous cerasomes (△, SPP_N = 0.595) in water with their corresponding SPP_N values derived from the fit;† e) Anisotropy of fluorescence of **RhB** dissolved in water and encapsulated in non-porous cerasomes (λ_{exc} = 510 nm).

† Note that a correlation of the solvent-related absorption and fluorescence maxima with dielectric continuum/reaction-field theory of solvation, relying on permittivity and refractive index, does not yield meaningful results. Even an exclusive correlation with the refractive index for the influence of dispersive forces, as has been reported for polymethine dyes which are usually only weakly solvatochromic, does not yield acceptable fits in this case.

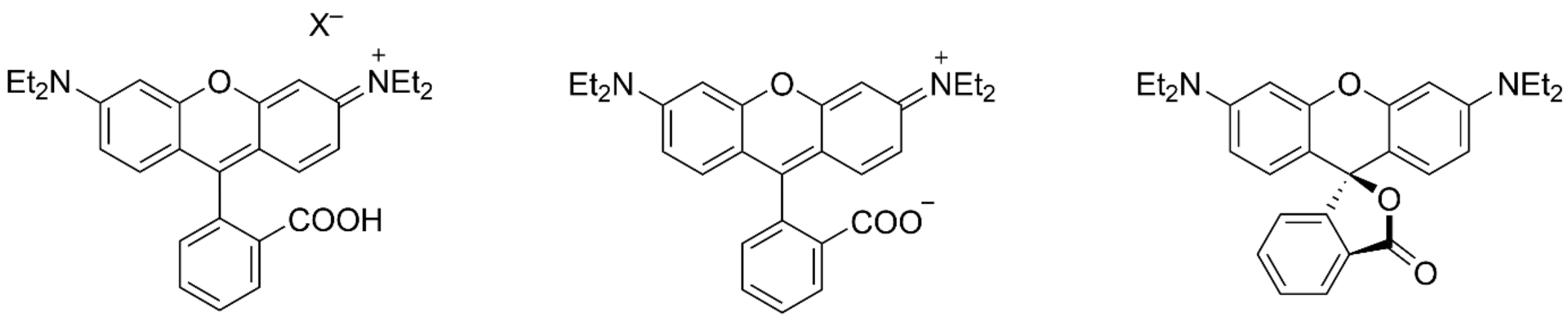

X = Cl, ClO_4, etc.

RhB_A **RhB_B** **RhB_L**

Figure S11. Prevalent forms of **RhB** in solution: intrinsically cationic acid form **RhB_A**, always being accompanied by a counteranion, mostly chloride or perchlorate (left), zwitterionic base form **RhB_B** and neutral lactone form **RhB_L**.

IV. Preparation and characterisation of porous cerasomes

Preparation of porous cerasomes

A fraction of 3 mg of lipid **3** was thawed and dissolved in 300 µL of ethanol. This solution was divided in 6 aliquots and ethanol was evaporated with argon to afford a thin layer of lipid on the Eppendorf vial's wall. In a next step, the following reagents were added into the vial: 600 µL of Milli-Q water at pH 3 (HCl), 100 µL of a solution of poloxamer (Pluronic P-123 (MW ≈ 5800 g mol^{-1}), Pluronic F-127 (MW ≈ 12600 g mol^{-1}) or Pluronic F-68 (MW ≈ 8350 g mol^{-1}) at 33.4 g L^{-1} in Milli-Q water at pH 3 (HCl). In addition, 10 µL of Fe_2O_3 nanoparticles (IONPs) at 6% in water and 300 µL of **R6G** at 1 g L^{-1} in Milli-Q water at pH 3 (HCl) were added, with the aim to accelerate the formation of the porous cerasomes. (Note that IONPs and **R6G** were only added for synthetic purposes as described recently by others, [12, 13] not to endow the cerasomes with magnetic or fluorescence properties. Both auxiliary agents were completely removed during post-synthetic clean-up.) This solution was mixed and sonicated following the same protocol as for non-porous cerasomes. The solution was left in the refrigerator for 4–14 d to let the formation of cerasomes with inclusions of poloxamers in the membrane occur. Poloxamers were removed by addition of 200 µL of HCl (1 M) in EtOH. The resulting solution was stirred for 40 min at room temperature, then EtOH was partially evaporated by flushing argon and the solution basified to obtain a pH above 10, stabilizing the cerasome membrane by full crosslinking. SEC was performed to clean the cerasomes from the dissolved poloxamer, residual lipid, **R6G** and IONPs.

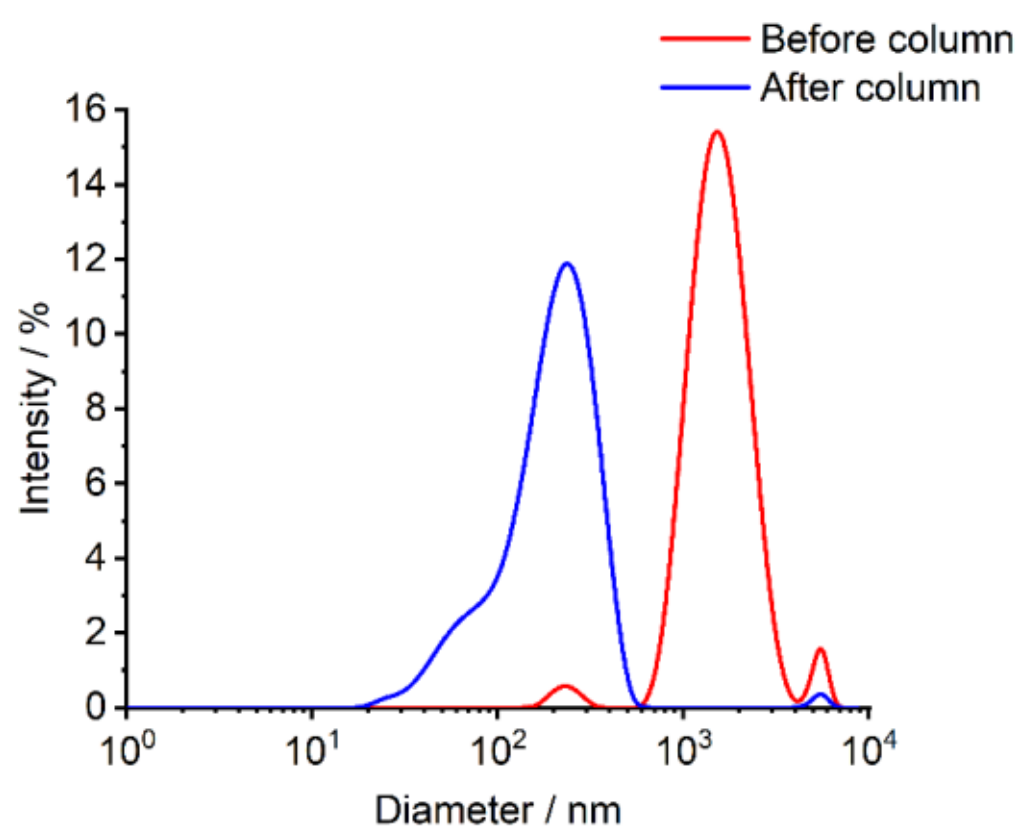

Figure S12. Typical graph of size measurements with DLS obtained after basification of the porous cerasome suspension before (red) and after (blue) size exclusion chromatography.

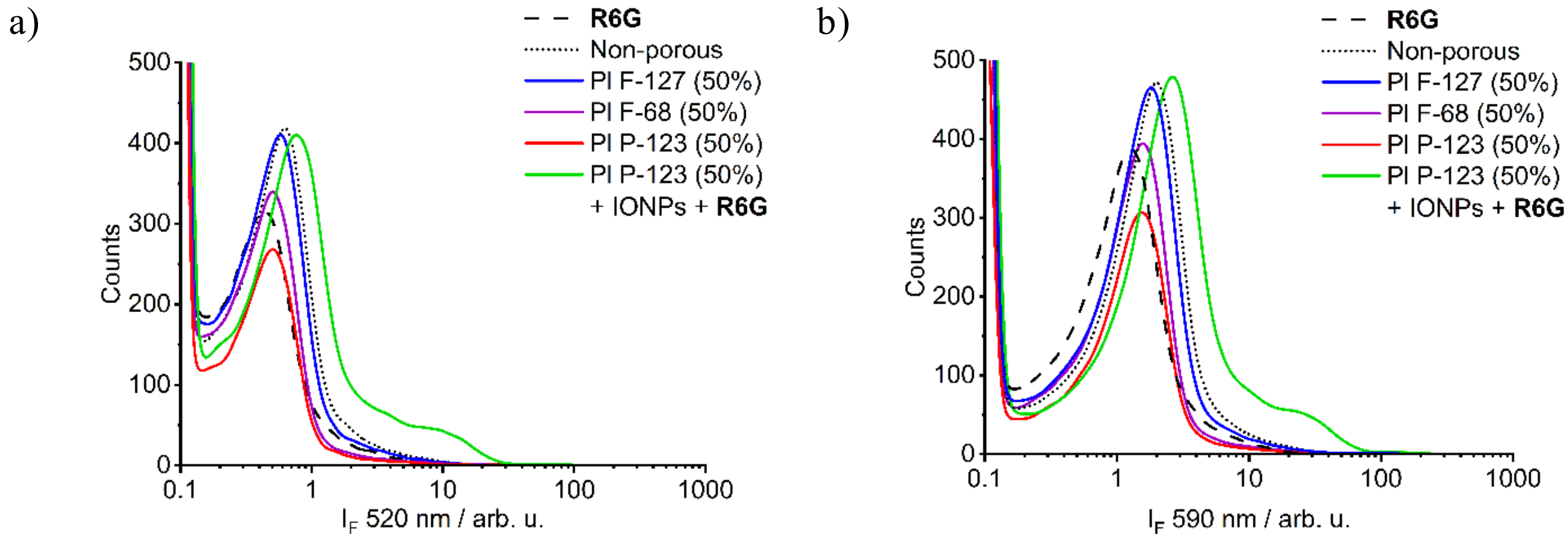

Figure S13. Histogram of fluorescence at 520 nm a) and 590 nm b) of a solution of **R6G** alone (0.25 g L^{-1}, dashed line) or in presence of non-porous cerasomes (black dots), cerasomes prepared with Pluronic® F-127 at 50% (blue), Pluronic® F-168 at 50% (purple), Pluronic® P-123 at 50% (red), or Pluronic® P-123 at 50% associated with IONPs and **R6G** (cerasomes solutions cleaned by SEC beforehand).

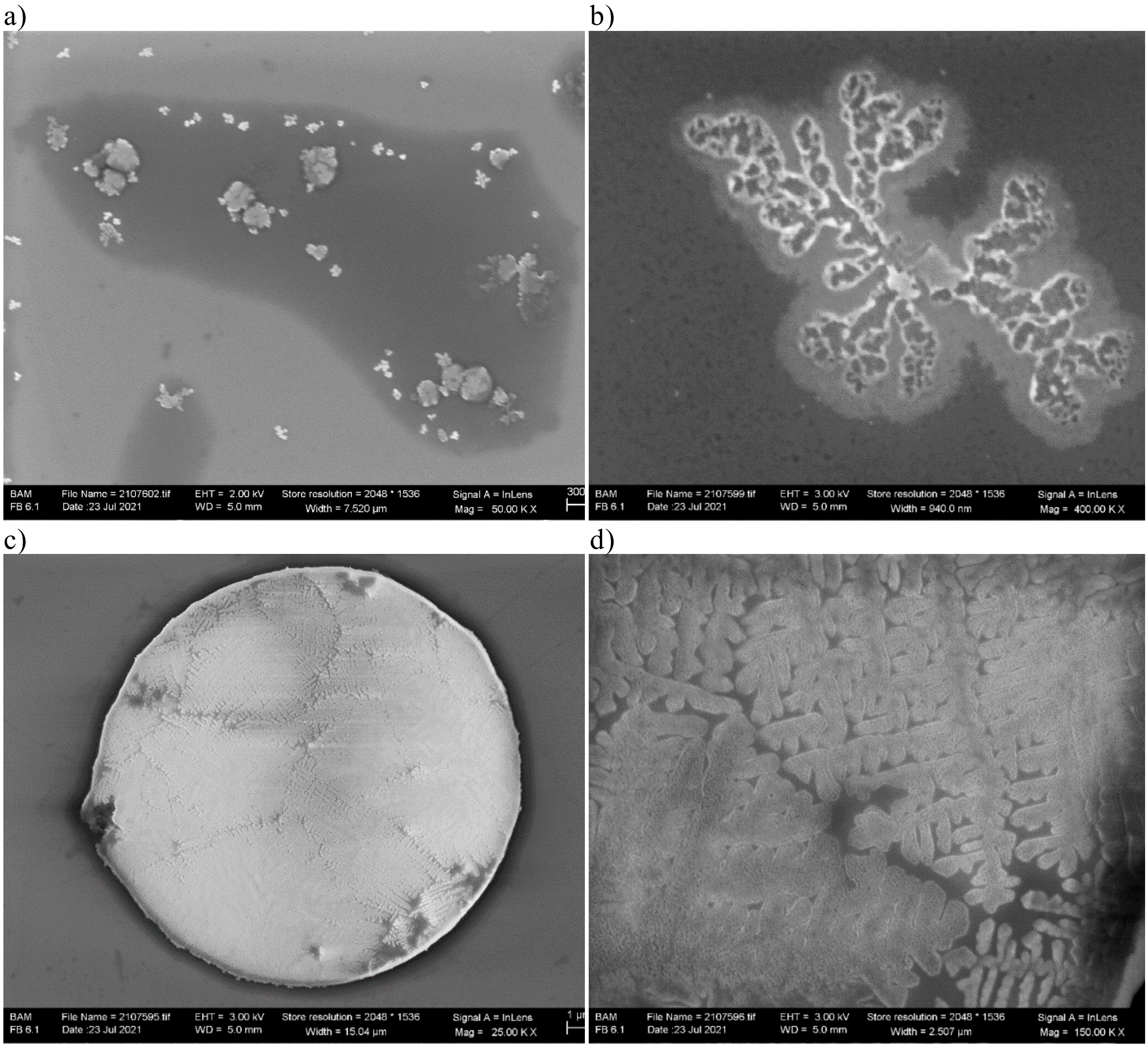

Figure S14. SEM images of a) porous cerasomes with sizes of 302 ± 60 nm; b) a destroyed porous cerasome due to the vacuum and irradiation conditions upon focusing; c) view of a big porous cerasome (10 µm) with d) a zoom on its surface.

Preparation of dye-loaded porous cerasomes through post-encapsulation and addition of gates

To prepare several porous cerasomes containing different dyes as reporters inside of their core, 1 mL of a solution of porous cerasomes was mixed at room temperature with 300 µL of a dye (**R6G**, **RhB**, **SRB**, **SfrO**, **RBP**, **NB**, **6-FAM**, **FITC4000**) at 4 g L^{-1} or of **FITC70000** for 24 h at 40 g L^{-1}. Subsequent addition of 25 µmol (6.5 µL) of *N'*-(3-trimethoxysilyl) propyl diethylenetriamine (**N3**) with reaction times of 12 , 3 and 1 h led to the attachment of polyamine groups at the cerasomes' surface, allowing the formation of pH- and ATP-controlled gates. SEC was performed with pH 3-buffered water to clean the cerasomes from residual **N3** and excess of dye using PD10 columns.

a)
b)

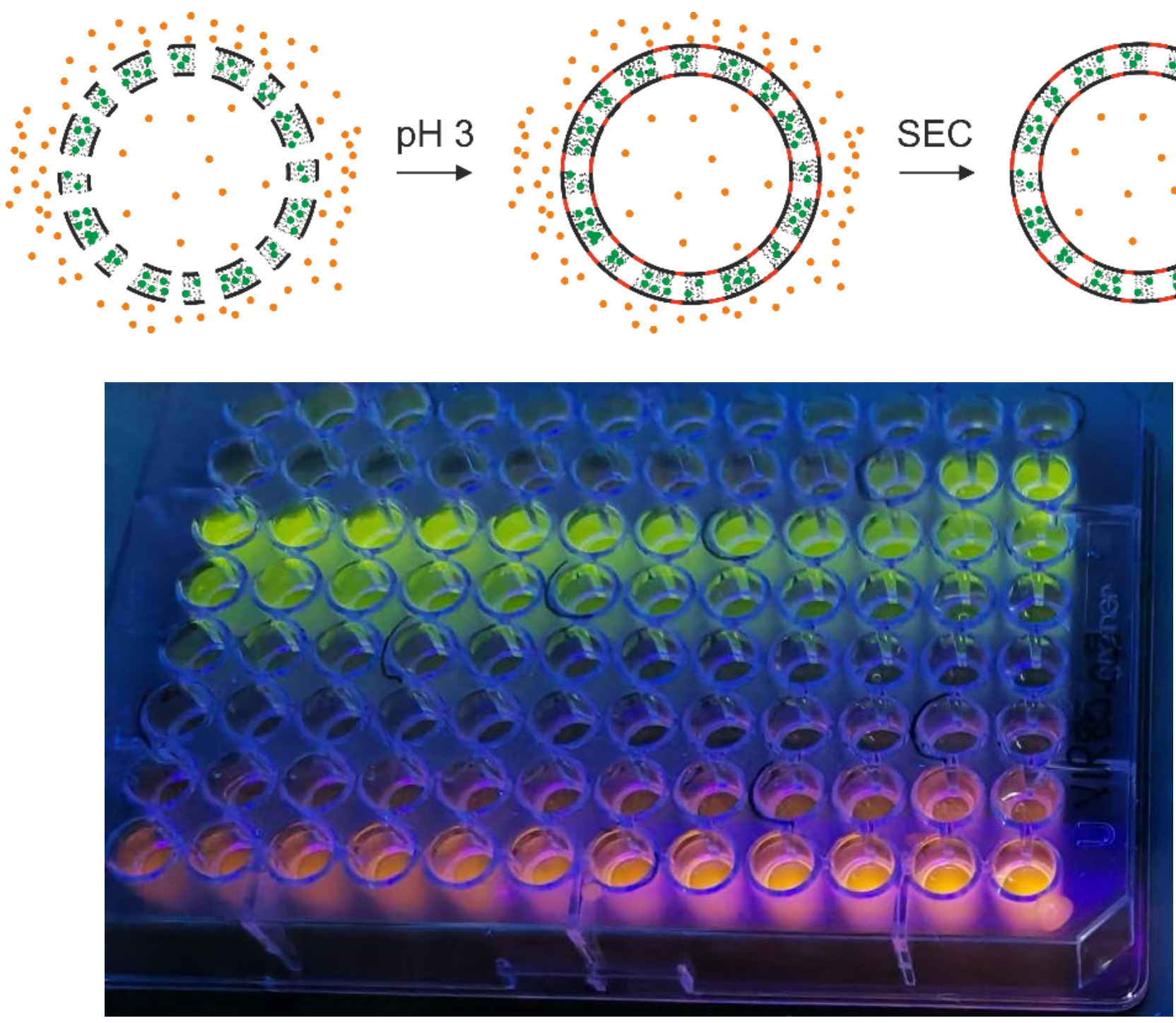

Figure S15. a) Separation of **RhB** and porous cerasomes loaded with **RhB** after gate closing through SEC; b) ELISA plate containing the washing fractions from SEC with **RhB**-loaded cerasomes (greenish fluorescence) in the early and free **RhB** (reddish fluorescence) in the later wells; photo taken under the UV-lamp (λ_{exc} = 365 nm).

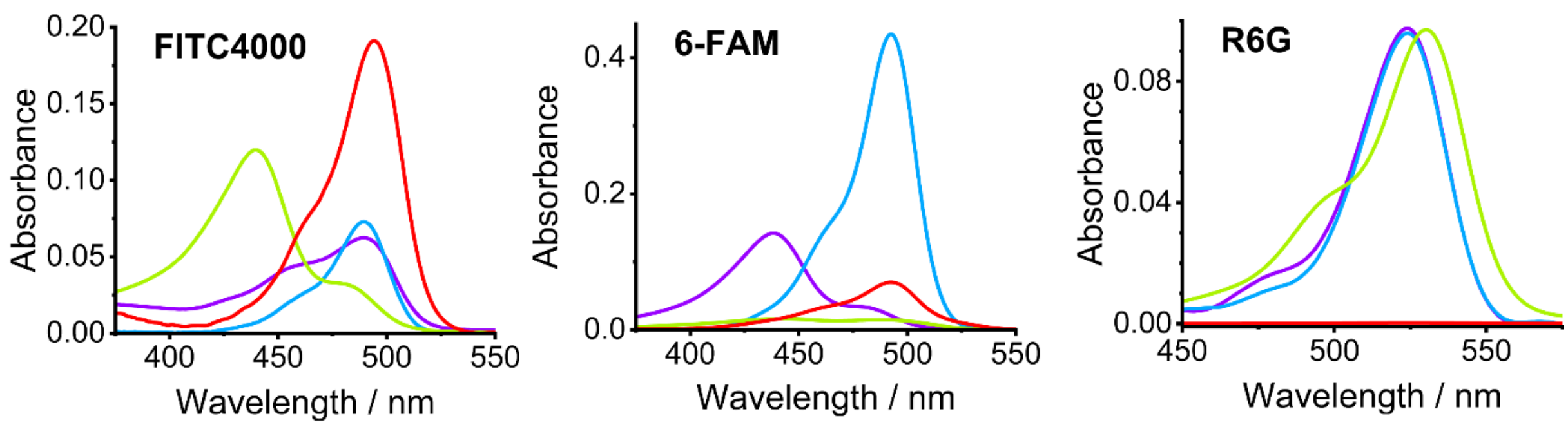

Figure S16. Absorption spectra of a solution containing dye-loaded porous cerasomes with pH responsive gates upon basification of the medium (purple line: non-porous cerasomes at pH 3, blue line: non-porous cerasomes at pH 9, green line: porous cerasomes at pH 3, red line: porous cerasomes at pH 9).

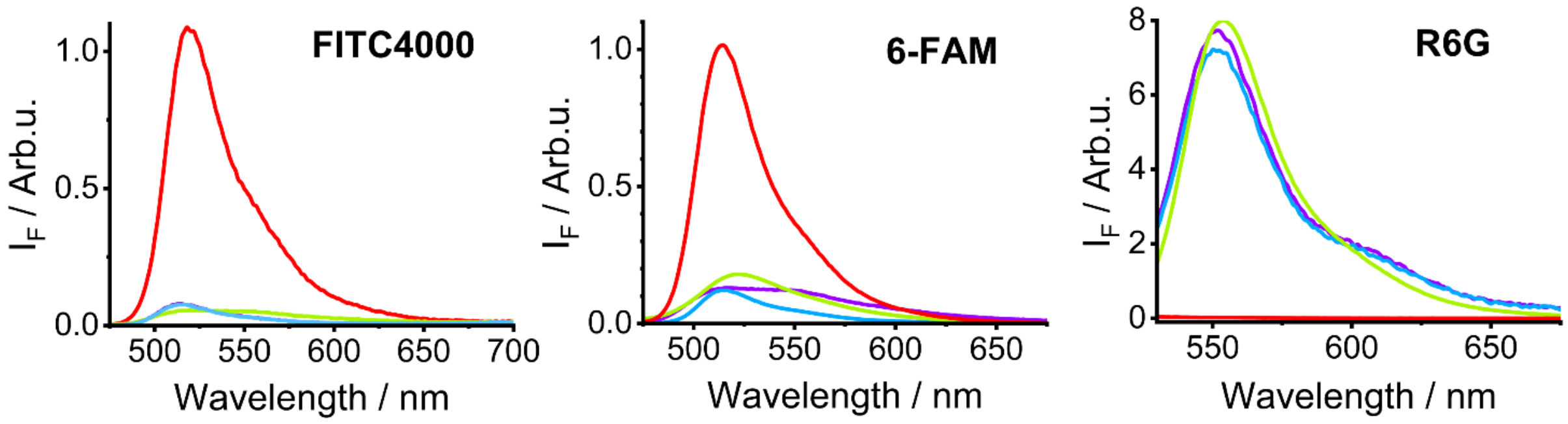

Figure S17. Corrected emission spectra of a solution containing dye loaded porous cerasomes with pH responsive gates upon basification of the medium (purple line: non-porous cerasomes at pH 3, blue line: non-porous cerasomes at pH 9, green line: porous cerasomes at pH 3, red line: porous cerasomes at pH 9).

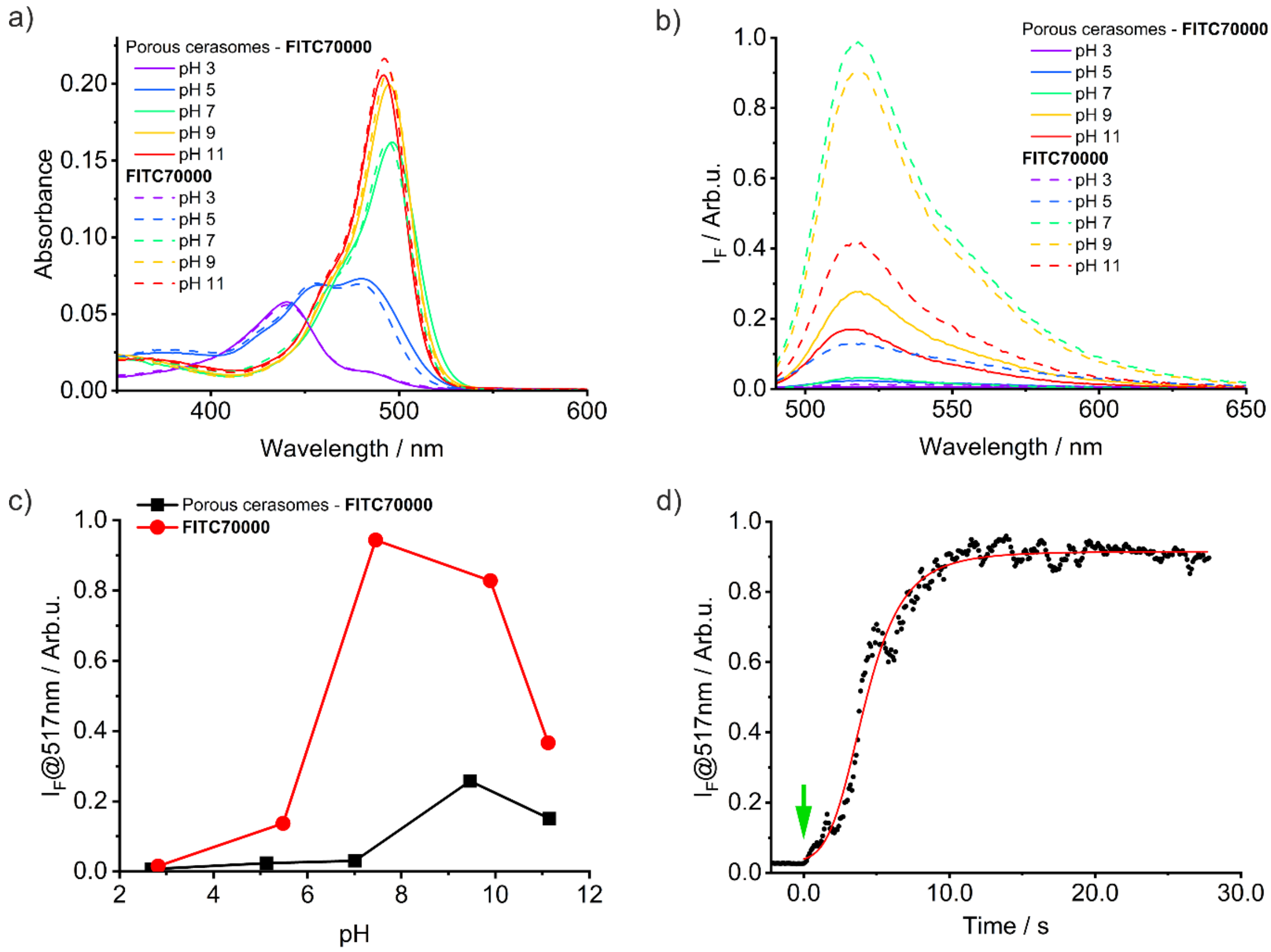

Figure S18. a) Absorption and b) emission spectra of porous cerasomes loaded with **FITC70000** and gated with **N3** and **FITC70000** in water at various pH; c) Corresponding emission intensities at 517 nm depending on the pH and d) emission intensity at 517 nm of **FITC70000**-loaded cerasomes upon switching from pH 3 to pH 9 (green arrow indicates addition of NaOH to the particles suspension) (λ_{exc} = 480 nm).

V. Response to ATP

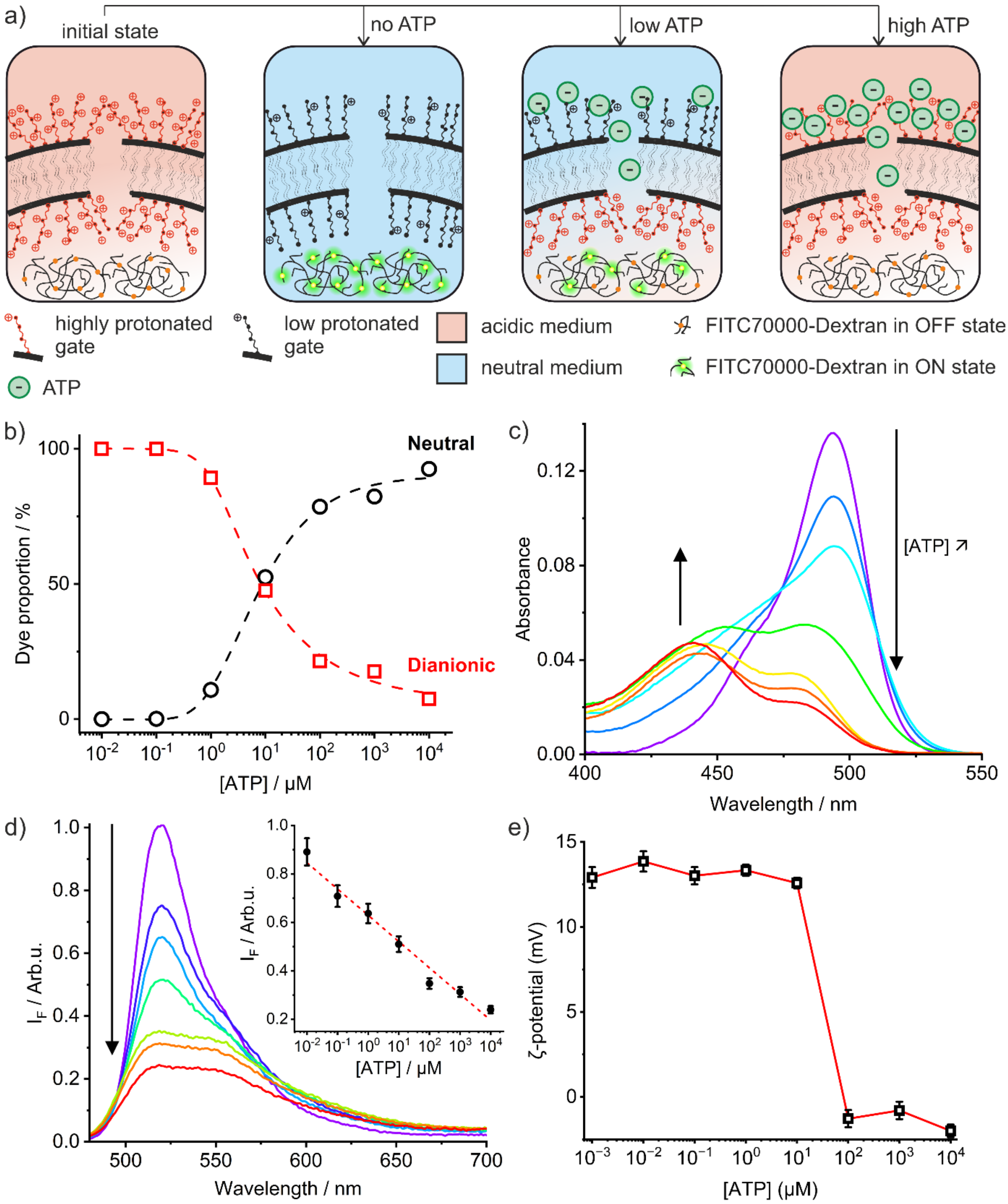

Figure S19. a) Scheme of the gated material in the initial state (as stored) in acidic medium and upon addition to MilliQ water in absence or presence of ATP allowing or preventing the opening of the pores and ATP in high concentration inducing a pH decrease; b) Calculated proportion of **FITC70000** dye in neutral or dianionic forms *vs* ATP concentration without buffer using the ratio of absorbance at 440 and 500 nm; c) Absorption and d) emission spectra of a solution containing **FITC70000**-loaded porous cerasomes with pH/anion responsive gates in basic media upon increasing concentration of ATP without buffer; inset in d): Calibration plot of sensory material response for ATP (λ_{exc} = 480 nm); e) Zeta potential of the suspension upon increasing concentration of ATP.

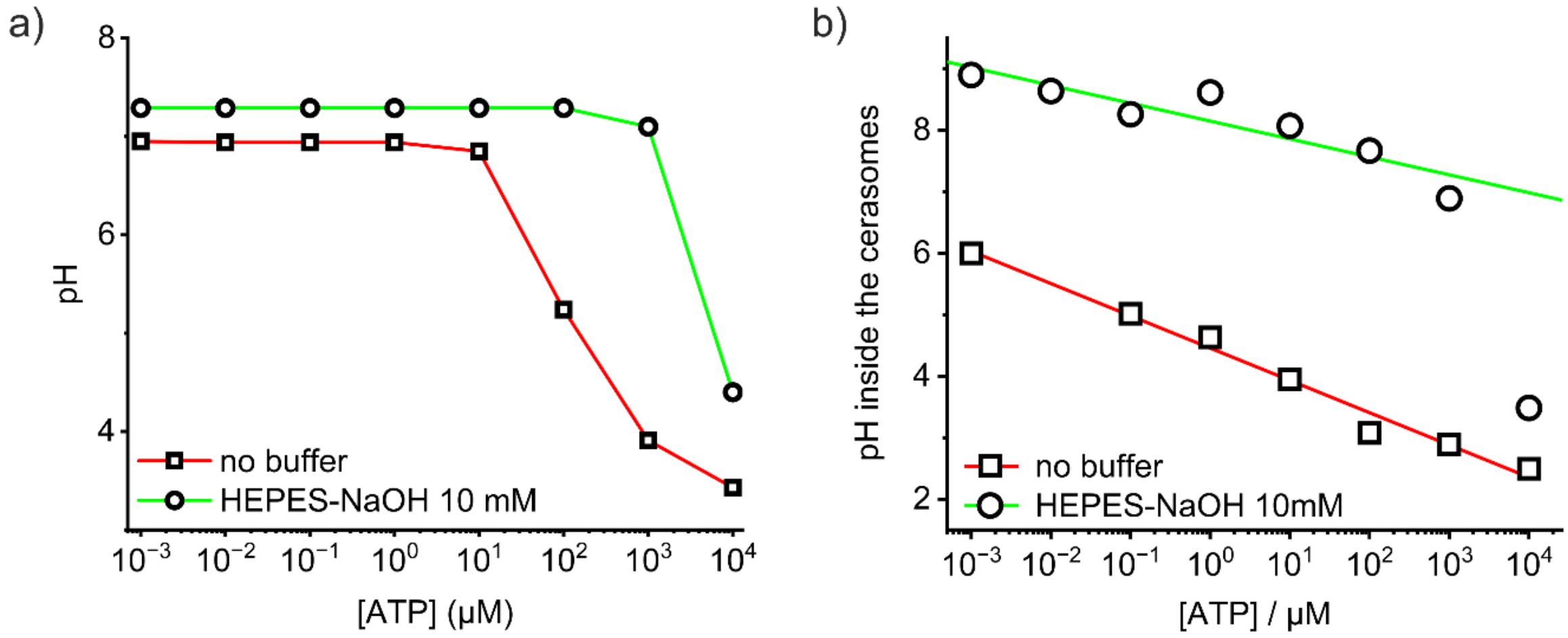

Figure S20. a) pH of the solution upon increasing concentration of ATP with and without HEPES-NaOH buffer (pH 7.3; 10 mM); b) Estimated pH inside the cerasome void calculated from the I_F/I_0 ratio *vs* ATP concentration with and without HEPES-NaOH buffer (pH 7.3; 10 mM) (linear fits; point at 10 mM excluded from the fit for data with buffer).

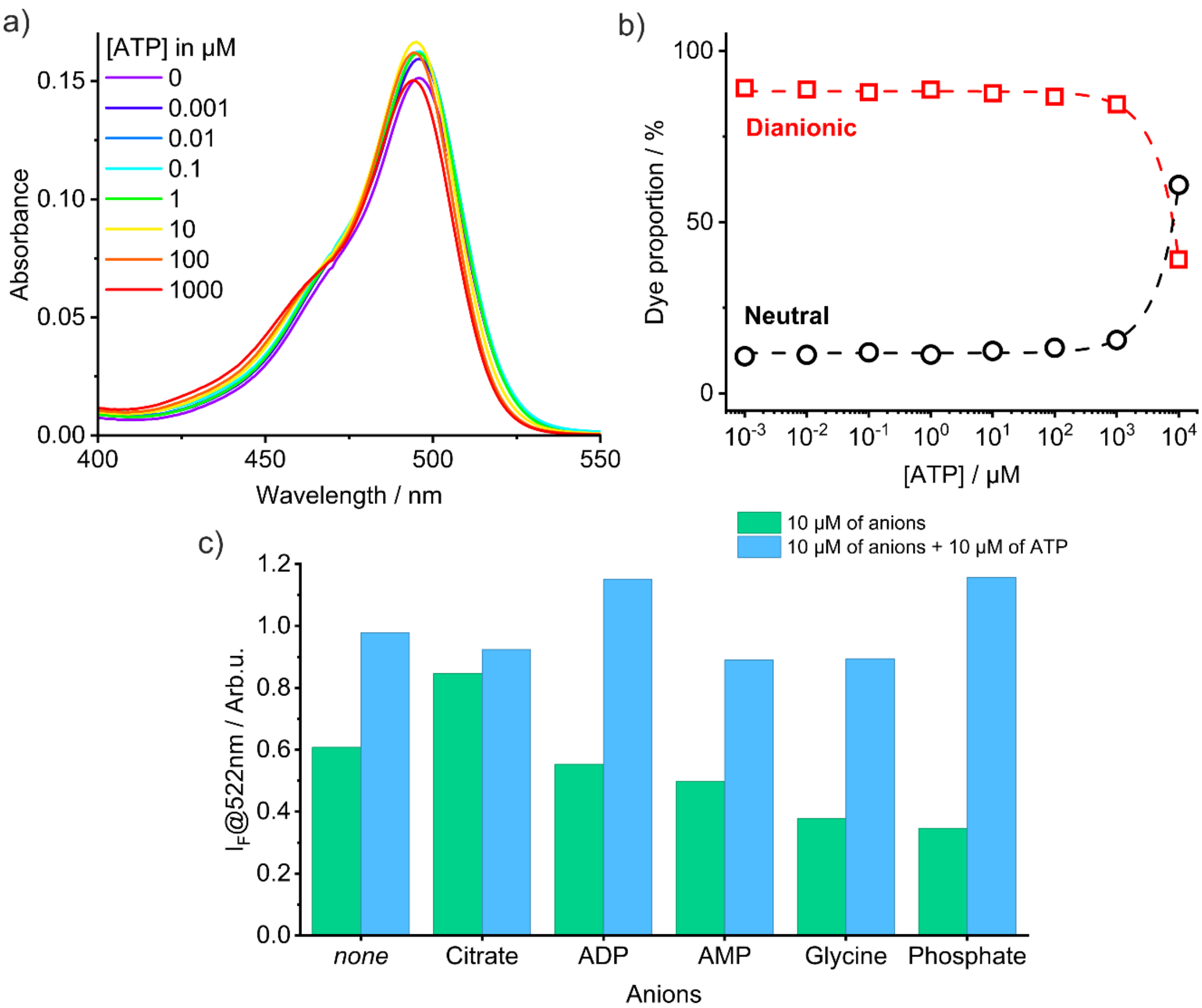

Figure S21. a) Absorption spectra of a solution containing **FITC70000**-loaded porous cerasomes with pH/anion responsive gates in HEPES-NaOH buffer (pH 7.3; 10 mM) upon increasing concentration of ATP; b) Calculated proportion of **FITC70000** dye in neutral or dianionic forms *vs* ATP concentration with HEPES-NaOH buffer (pH 7.3; 10 mM) using the ratio of absorbance at 440 and 500 nm; c) Response of the sensory particles towards diverse anions: citrate, adenosine diphosphate sodium salt (ADP), adenosine monophosphate sodium salt (AMP), phosphate and glycine before (green) and after (blue) addition of ATP (c_{anions} = 10 μM, λ_{exc} = 480 nm).

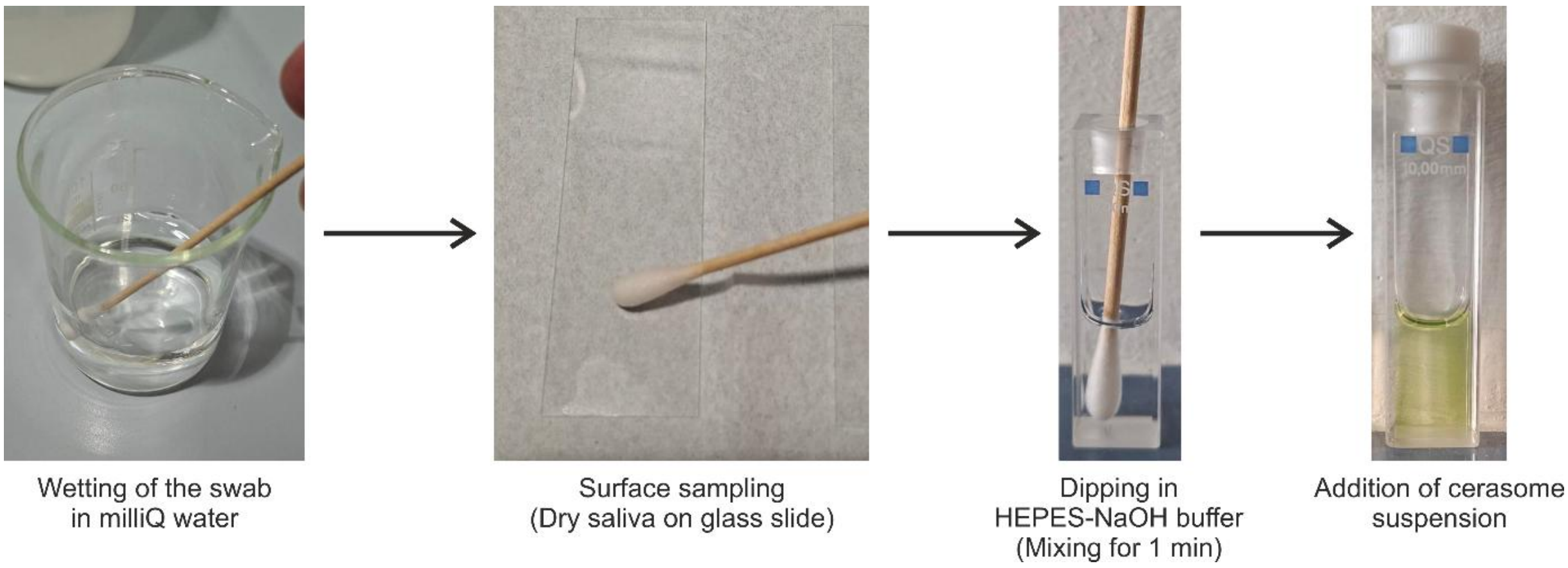

Figure S22. Swab sampling protocol for analysis of a glass surface contaminated with saliva with the **FITC70000**-loaded porous cerasomes with pH/anion responsive gates in HEPES-NaOH buffer (pH 7.3; 10 mM).

Table S2. Comparison of recently reported ATP fluorescent sensors

Entry	Sensor type	Recognition	LOD	Linear range	Response time	Application	Reference
#1	Lanthanide MOF	Luminescence quenching	20 µM	*n.r.**	Fast	no	[14]
#2	Aptamer-Au particles	Switch to molecular beacon (FRET)	3.35 nM	100 nM – 1 mM	~8 min	HeLa-cell treatment but not quantitative intracellular ATP monitoring	[15]
#3	Aptamer	Switch to molecular beacon (FRET)	125 nM	0 – 1 µM	Real-time monitoring	Cell-culture medium demonstrated only qualitative measurement	[16]
#4	Fluorescent protein	Conformational change alters FRET	100 µM	*n.r.*	Slow	ATP concentration monitoring in E. coli	[17]
#5	Fluorescent probe	Host-guest interaction	0.18 mM	1.5 – 5.0 mM	< 1 min	HeLa-cells treatment Quantitative monitoring in L02 cells In vivo assays	[18]
#6	N-doped graphene quantum dots	Competition with graphene for Fe^{3+} binding	1.16 nM	0 – 10 mM	Fast	4T1 cells and shrimps' treatment Portable hydrogel kits	[19]
#7	MOF	MOF collapse and decreased fluorescence	7.06 µM	0 – 100 µM	1 min	Extracellular ATP monitoring in plants	[20]
#8	PEI-Coumarin fluorescent receptor	Host–guest interaction	*n.r.*	0 – 1 µM	*n.r.*	no	[21]
#9	Aptamer	Competition assay with Ag nanoclusters	0.27 nM	5 nM – 10 µM	> 7 h	Quantification in human serum	[22]
#10	pH responsive cerasomes	Electrostatic interaction	20 nM	20 nM – 1 mM	< 30 s	Swab test for hygiene monitoring	*This article*

* *n.r.: not reported*

VI. References

1. K. Katagiri; M. Hashizume; K. Ariga; T. Terashima; J. Kikuchi, "Preparation and characterization of a novel organic-inorganic nanohybrid "cerasome" formed with a liposomal membrane and silicate surface", *Chemistry* 13, (2007), 5272–5281. https://doi.org/10.1002/chem.200700175.

2. E. Climent; M. Hecht; K. Rurack, "Loading and Release of Charged and Neutral Fluorescent Dyes into and from Mesoporous Materials: A Key Role for Sensing Applications", *Micromachines* 12, (2021), 249. https://doi.org/10.3390/mi12030249.

3. K. Rurack, Fluorescence Quantum Yields: Methods of Determination and Standards. In *Standardization and Quality Assurance in Fluorescence Measurements I: Techniques*, U. Resch-Genger, Ed. Springer: Berlin, 2008; Vol. 05, pp 101–145.

4. R. Sjöback; J. Nygren; M. Kubista, "Absorption and fluorescence properties of fluorescein", *Spectrochimica Acta Part a-Molecular and Biomolecular Spectroscopy* 51, (1995), L7–L21. https://doi.org/10.1016/0584-8539(95)01421-p.

5. F. López Arbeloa; T. López Arbeloa; E. Gil Lage; I. López Arbeloa; F. C. De Schryver, "Photophysical properties of rhodamines with monoethylamino group R19 and R6G in water-ethanol mixtures", *Journal of Photochemistry and Photobiology a-Chemistry* 56, (1991), 313–321. https://doi.org/10.1016/1010-6030(91)80031-C.

6. S. Jonnalagadda; N. Gollapalli, "Nonlinear kinetics and mechanism of nile blue reaction with acidic bromate and with aqueous bromine", *South African Journal of Chemistry* 54, (2001), 41 – 59. https://doi.org/10.10520/EJC21944.

7. H. P. M. Oliveira; A. J. Camargo; L. G. Macedo; M. H. Gehlen; A. B. F. da Silva, "Synthesis, structure, electronic and vibrational spectra of 9-(Diethylamino)-benzo(a)phenoxazin-7-ium-5-N-methacrylamide", *Spectrochimica Acta Part A: Molecular and Biomolecular Spectroscopy* 58, (2002), 3103–3111. https://doi.org/10.1016/S1386-1425(02)00119-1.

8. C. D. Borsarelli; S. G. Bertolotti; C. M. Previtali, "Thermodynamic changes in the photoinduced proton-transfer reaction of the triplet state of safranine-T", *Photochemical & Photobiological Sciences* 1, (2002), 574–580. https://doi.org/10.1039/B202683J.

9. K. Kalyanasundaram, "Photophysics, photochemistry and solar-energy conversion with tris(bipyridyl)ruthenium(II) and its analogs", *Coordination Chemistry Reviews* 46, (1982), 159–244. https://doi.org/10.1016/0010-8545(82)85003-0.

10. J. Catalán; V. López; P. Pérez; R. Martín-Villamil; J. G. Rodríguez, "Progress towards a generalized solvent polarity scale - the solvatochromism of 2-(dimethylamino)-7-nitrofluorene and its homomorph 2-fluoro-7-nitrofluorene", *Liebigs Annalen*, (1995), 241–252. https://doi.org/10.1002/jlac.199519950234.

11. J. R. Lakowicz, Corrected Emission Spectra. In *Principles of Fluorescence Spectroscopy*, 3rd ed.; Springer: New York, 2006; pp 52–54.

12. D. R. Khan; E. M. Rezler; J. Lauer-Fields; G. B. Fields, "Effects of Drug Hydrophobicity on Liposomal Stability", *Chemical Biology & Drug Design* 71, (2008), 3–7. https://doi.org/10.1111/j.1747-0285.2007.00610.x.

13. P. B. Santhosh; B. Drasler; D. Drobne; M. E. Kreft; S. Kralj; D. Makovec; N. P. Ulrih, "Effect of superparamagnetic iron oxide nanoparticles on fluidity and phase transition of phosphatidylcholine liposomal membranes", *International Journal of Nanomedicine* 10, (2015), 6089–103. https://doi.org/10.2147/IJN.S89679.

14. P. Zhao; Y. Liu; C. He; C. Duan, "Synthesis of a Lanthanide Metal-Organic Framework and Its Fluorescent Detection for Phosphate Group-Based Molecules Such as Adenosine Triphosphate", *Inorganic Chemistry* 61, (2022), 3132–3140. https://doi.org/10.1021/acs.inorgchem.1c03412.

15. Y. Lin; X. Tao; S. Gao; N. Li; Z. Dai, "Highly sensitive and stable fluorescent aptasensor based on an exonuclease III-assisted amplification strategy for ATP detection", *Analytical Biochemistry* 675, (2023), 115210. https://doi.org/10.1016/j.ab.2023.115210.

16. A. Bacon; O. Alkhamis; C. Byrd; J. Perry; J. Canoura; Y. Xiao, "Reagentless Real-Time ATP Monitoring with New DNA Aptamers", *Small* 21, (2025), e08898. https://doi.org/10.1002/smll.202508898.

17. J. Deng; Y. Hou; R. Gong; M. Chen; P. Peng; D. Men; X. E. Zhang, "A Red Fluorescent Genetically Encoded Biosensor for the Visualization of ATP in Live Cells", *ACS Sensors* 11, (2026), 3793–3805. https://doi.org/10.1021/acssensors.5c04623.

18. J. Lu; K. Dou; Z. Geng; Z. Wang, "Construction of near infrared ratiometric fluorescent probes with emission crosstalk-free for reversible ATP tracking in mitochondria and tumors", *Sensors and Actuators B: Chemical* 390, (2023). https://doi.org/10.1016/j.snb.2023.134043.

19. H. Zhang; J. Wang; S. Wei; C. Wang; X. Yin; X. Song; C. Jiang; G. Sun, "Nitrogen-doped graphene quantum dot-based portable fluorescent sensors for the sensitive detection of Fe(3+) and ATP with logic gate operation", *Journal of Material Chemistry B* 11, (2023), 6082–6094. https://doi.org/10.1039/d3tb00327b.

20. Q. Qiu; S. Sun; H. Yuan; S. Zhang; Y. Feng; F. Wang; Y. Zhu; M. Zhou; Y. Wang, "Second near-infrared fluorescent Metal-Organic framework sensors for in vivo extracellular adenosine triphosphate monitoring", *Biosensors and Bioelectronics* 251, (2024), 116114. https://doi.org/10.1016/j.bios.2024.116114.

21. E. Delgado-Pinar; M. Medeiros; T. Costa; J. S. Seixas de Melo, "Highly Selective Fluorescent Sensors: Polyethylenimine Derivatives of Triphenylamine and Coumarin for GTP and ATP Interaction via Fluorescence Lifetime Imaging Microscopy", *ACS Applied Polymer Materials* 5, (2023), 6176–6185. https://doi.org/10.1021/acsapm.3c00834.

22. S. Cai; X. Chen; H. Chen; Y. Zhang; X. Wang; N. Zhou, "A fluorescent aptasensor for ATP based on functional DNAzyme/walker and terminal deoxynucleotidyl transferase-assisted formation of DNA-AgNCs", *Analyst* 148, (2023), 799–805. https://doi.org/10.1039/d2an02006h.
